# Modelling in-hospital length of stay: A comparison of linear and ensemble models for competing risk analysis

**DOI:** 10.1371/journal.pone.0322101

**Published:** 2025-08-26

**Authors:** Juan Carlos Espinosa-Moreno, Fernando García-García, Naia Mas-Bilbao, Susana García-Gutiérrez, María José Legarreta-Olabarrieta, Dae-Jin Lee

**Affiliations:** 1 Basque Center for Applied Mathematics (BCAM), Bilbao, Basque Country, Spain; 2 Department of Mathematics, University of the Basque Country (UPV/EHU), Leioa, Basque Country, Spain; 3 Galdakao-Usansolo University Hospital, Critical Care Unit, Galdakao, Basque Country, Spain; 4 Galdakao-Usansolo University Hospital, Research Unit, Galdakao, Basque Country, Spain; 5 Network for Research on Chronicity, Primare Care, and Health Promotion (RICAPPS), Madrid, Spain; 6 Biosistemak, Barakaldo, Bizkaia, Spain; 7 School of Science and Technology, IE University, Madrid, Madrid, Spain; State University of New York at Oswego UNITED STATES OF AMERICA

## Abstract

Length of Stay (LoS) for in-hospital patients is a relevant indicator of efficiency in healthcare. Moreover, it is often related to the occurrence of hospital-acquired complications. In this work, we aim to explore time-to-event analysis for modelling LoS. We employed competing risk models (CR), as we considered two mutually exclusive outcomes: favorable discharge and deterioration. The explanatory variables included the patient’s sex, age, and longitudinal vital signs collected from a dataset comprising N=19,602 admissions. To address sparse measurements, we transformed longitudinal vital signs into cross-sectional statistics. Our approach involves data pre-processing, imputation of missing data, and variable selection. We proposed four types of CR models: Cause-specific Cox, Sub-distribution hazard, and two variants of Random Survival Forests, with both generalised Log-Rank test (cause-specific hazard estimates) and Gray’s test (cumulative incidences estimations) as node splitting rules. Performance in LoS CR models was evaluated over a time frame from 2 to 15 days. Additionally, we considered baselines with two well-established clinical early warning scores the National Early Warning Score (NEWS) and the Modified Early Warning Score (MEWS). The best model was Random Survival Forest using Gray’s test split, with Integrated Brier Score[×100] of 0.386, C-Index above 99%, and Brier Score below 0.006, along the entire time frame. Employing cross-sectional statistics derived from vital signs, along with rigorous data pre-processing, outperformed the degree of correctness of modelling LoS, compared to NEWS and MEWS.

## Introduction

In the context of hospitalisation, Length of Stay (LoS) denotes the time that each patient spends from admission to their endpoint. Consequently, LoS is widely used to assess the efficiency and sustainability of healthcare [1–3]: Unnecessarily long stays may lead to increased hospital-acquired complications (e.g., healthcare-associated infections, falls, medication errors or delirium) and high costs [4]. LoS serves as a suitable indicator of efficiency in resource management: e.g. bed capacity, staffing, equipment, medication usage or patient turnover rates [5]. Furthermore, LoS is important for evaluating hospital quality, productivity, and overall performance [6]. Characterising which source of demographic and clinical information best explains LoS can contribute to improving resource planning, capacity management, and staff optimisation, resulting in increased patient access, enhanced safety, reduced healthcare costs, and most efficient resource utilisation [7].

Estimations of LoS by human experts (the prevailing standard of care) entail reliability issues: omission of the patients’ background information [8], the accuracy of predictions depending on physicians’ expertise, and other issues such as treatment complications that prolong LoS [9]. This has motivated research on the use of automatic tools to assess LoS [7,10].

Various statistical regression techniques have been employed to model LoS in different medical contexts. [11] used gamma mixture regression in childbirth. [12] proposed lognormal-based mixture models to fit LoS, applying them to data from medical and surgical intensive care units (ICUs). [13] recommended mixed-exponential and phase-type distributions for stroke-related patients LoS. [14] used multiple linear regression (MLR) for the prediction of LoS in pediatric emergency department (ED) patients, whereas [15] applied MLR for in-hospital patients’ LoS.

Machine learning (ML)-based regression techniques have also been applied to model and predict LoS. In [16], twenty-nine ML algorithms were trained to predict LoS in patients who underwent craniotomy for brain tumors. In [7], characteristics of individuals in in-patient departments served as inputs for various regression models, including MLR, K-Nearest neighbors, decision trees (DTs), random forest (RF), artificial neural networks (ANNs), and eXtreme gradient boosting (XGBoost).

Several studies have also proposed ML-classification techniques: First, they binned LoS (most of them dividing it into two categories short-term vs long-term categories); subsequently, using ML-based binary prediction. [17] analysed LoS for ICU patients following cardiac surgery via logistic regression. [18] modelled LoS for surgical patients, comparing Support Vector Machine (SVM), MLR, and ANNs. [19] applied a stacked-ensemble method for diabetic patients. Other studies, such as [20], binned LoS into three categories for cardiac patients, and applied RF, ANN, SVM, and Bayesian Networks for prediction; [21] binned LoS into five groups, using SVM, deep neural networks, RF, XGBoost, and DTs for prediction in an ED.

Survival analysis has also been proposed to analyse LoS, although to a lesser extent. [22] proposed Cox proportional hazards and parametric models for LoS in an inpatient unit. [23] modelled LoS for patients with burns, using a competing risk cause-specific hazard model, with two possible endpoints: decease and discharge. [24] used competing risk-accelerated failure time for ED patients, with three possible endpoints: discharge, admission, and decease.

Regarding the input data, authors often incorporated covariates. The most frequently used are demographics (e.g., sex, age), medical attributes (e.g., comorbidities, diagnosis, severity, laboratory information, medical tests, triage level, diseases, acuity), and healthcare characteristics (e.g., ward round notes, in-hospital procedures, hospital admission). Other less frequent covariates are social (e.g., marital status) and socioeconomic information (e.g., insurance details). Nevertheless, only a limited number of studies use vital signs in modelling LoS. These data, commonly housed within Electronic Health Records (EHRs), play an important role in the development of Early Warning Systems (EWS), which are designed to identify and assess clinical deterioration in patients [25–30].

Out of the ICU, vital signs tend to be recorded with low frequency (often once every 8 to 24 hours) and at irregular time intervals. Moreover, interventions provided to patients and manual assessment of certain variables contribute to the complexity of recording vital signs [27,31].

Some studies, such as [32], have shown that the time between vital signs monitoring in patients with lower emergency severity index values was increased. Additionally, missing recordings can sometimes be attributed to random issues, such as data entry errors or occasional lapses in recording [33].

These issues pose a major modeling challenge when using longitudinal measurements as covariates. To address this challenge, a commonly successful strategy is to analyze trends and calculate cross-sectional statistics from the longitudinal data. This approach simplifies the data by converting longitudinal measurements into a form that is easier to analyze in models that do not inherently handle longitudinal data, as suggested by [25–27,29,31,34].

This article aims to present a workflow for modelling LoS as a function of vital signs. The proposed steps are: First, to calculate cross-sectional statistics from longitudinal vital signs. Second, stratify the data into random partitions. Third, impute missing data and apply variable selection techniques to avoid potential collinearity between covariates. Fourth, use competing risks (CR) in LoS analysis. Finally, to analyse the contribution of different covariates to aid clinical interpretation. This approach, to the best of our knowledge, has not been yet used with vital sign cross-sectional statistics as covariates, in conjunction with the proposed data pre-processing.

The remainder of the paper is organised as follows: The “Materials and methods” section describes the data pre-processing and the models proposed for missing data imputation, variable selection, and competing risks; “Results” provides a statistical description of the data and details the models’ performance; whereas “Discussion” highlights the main results, strengths, and limitations of this work. Finally, “Conclusions” summarises the main results and contributions.

## Materials and methods

### Study design

A retrospective observational cohort study was conducted at the Galdakao-Usansolo University Hospital (GUUH), a tertiary hospital in Bizkaia (Basque Country, Spain). As an inclusion criterion, we collected the pseudonymized EHRs of all hospitalisation episodes with admission dates in the year 2019, for stays lasting at least 24 hours of duration. An episode refers to a continuous period during which a patient is admitted to and receives care in a hospital. It begins with the patient’s admission and ends when they reach any of the possible endpoints (e.g. favourable discharge).

Each hospitalisation episode had only one of two possible endpoints: either a favourable discharge (i.e. without complications), or a deterioration endpoint. Following the care practices at GUUH, deterioration is a composite outcome that includes non-pre-scheduled (≥12h) stays in either ICU, respiratory ICU, the stroke unit, or in-hospital death.

Aligned with the practical interests of GUUH clinicians, the endpoint corresponding to a certain episode is defined as the event that takes place first: either a favorable discharge from the hospital, or any circumstance of deterioration (ICU admission, death, etc. as defined by our clinical team’s criteria). Note that these two endpoints are collectively exhaustive –i.e. its union equals the space of all possible events–, as well as mutually exclusive: we only consider the first event to occur, no further information is considered afterwards: e.g. for our analyses, for an episode in which the patient spent time in a non-prescheduled ICU stay, it is irrelevant for us here an eventual favourable discharge or a decease, since only the first chronological endpoint is studied. This ensures no overlap between episode endpoints, hence mutually exclusive. We also acknowledge that this is, to some extent, an ‘ad hoc’ criterion, as different studies may use alternative definitions based on their specific contexts or datasets.

Noteworthy, in the uncommon circumstance that a patient should be re-admitted within the first 48 hours immediately after a favorable discharge from a previous episode of his/hers, then our clinicians instructed that -according to the GUUH hospital protocols-, the second episode should be considered a prolongation of the first one, and thus both merged. Consequently, then the resulting endpoint was that corresponding to the latter sub-episode. No cases of third or extra re-admissions took place. For periods beyond 48 hours, episodes were deemed isolated and independent.

At the moment of the retrospective extraction of EHRs (date: 2022-03-28), all hospitalisations under consideration had already finished. With this retrospective data collection, we encountered that all episodes had already reached their natural endpoints. As a result, there was no right-censoring in the data, which tends to occur when a patient’s event status is unknown beyond a certain time point, typically due to the end of the study end or a loss of follow-up. Remarkably, since all hospitalisations in our dataset had been fully observed and reached its completion before data extraction, for every episode we could unambiguously determine its definitive outcome/endpoint, leaving no incomplete or censored records.

### Data description

Our dataset contains detailed information on the patient’s sex, age, and seven vital signs: body temperature, systolic and diastolic blood pressure (BP), heart rate, respiratory rate, peripheral oxygen saturation (SpO_2_), and level of consciousness. At GUUH, the current practice for recording the level of consciousness is to use an in-house variation of the standard AVPU scale [35], as follows: Level I for ‘Alert’, level II for both AVPU’s ‘Voice’ and ‘Pain’, and level III for ‘Unresponsive’. Whenever data about consciousness was absent, we used a new ‘Missing’ tag.

### Data pre-processing

Our clinical team established physiologically feasible ranges for each vital sign, detailed in the supplementary materials: Table A in S.A Data (S1 Appendix). Measurements out of range were marked as missing, assuming they were measurement or transcription error.

Vital signs data were structured using the ricu R package [36]. We aggregated measurements per hourly intervals; and if multiple measurements were originally recorded within the same hour, we summarised them by their median.

Let us consider the *i*-th hospitalisation episode, out of a total of *N* in our dataset. Let *n*_*i*_ be the number of time points at which vital sign x was recorded in that *i*-th episode. For measurements $x^{i}=\{x_{1}^{i},x_{2}^{i},\ldots ,x_{n_{i}}^{i}\}$ at times $t^{i}=\{t_{1}^{i},t_{2}^{i},\ldots ,t_{n_{i}}^{i}\}$, the cross-sectional statistics proposed are described in Table 1.

**Table 1:** Proposed cross-sectional statistics for vital signs longitudinal measurements.

Name	Formula	Remark
Mean	${\overset{―}{x}}^{i}=\frac{1}{n_{i}}\sum_{j=1}^{n_{i}}x_{j}^{i}$	Used in [27,31,34]
Maximum	$\max^{i}=\max(x_{1}^{i},x_{2}^{i},\ldots ,x_{n_{i}}^{i})$	Used in [25–27,29,31]
Minimum	$\min^{i}=\min(x_{1}^{i},x_{2}^{i},\ldots ,x_{n_{i}}^{i})$	Used in [25–27,29,31]
First observation	${\text{first}}^{i}=x_{1}^{i}$	Used in [26,29]
Last observation	${\text{last}}^{i}=x_{n_{i}}^{i}$	Used in [25,26,29]
Standard deviation	${\text{SD}}^{i}=\frac{1}{n_{i}-1}\sum_{j=1}^{n_{i}}{\left(x_{j}^{i}-{\overset{―}{x}}^{i}\right)}^{2}$	Used in [27,31,34]
Interquartile range	${\text{IQR}}^{i}=Q_{3}-Q_{1}$	*Q*_1_ and *Q*_3_ used in [34]
Coefficient of variation	${\text{CV}}^{i}=\frac{{\text{SD}}^{i}}{|{\overset{―}{x}}^{i}|}$	—
Average percentage change	${\text{APC}}^{i}=\frac{1}{n_{i}-1}\sum_{j=2}^{n_{i}}\frac{x_{j}^{i}-x_{j-1}^{i}}{x_{j-1}^{i}}$	Average change in the current value with respect to the previous, normalised by the previous. The numerator is also known as ‘delta’ change and used in [27]
Average change per time unit	${\text{ACPTU}}^{i}=\frac{1}{n_{i}-1}\sum_{j=2}^{n_{i}}\frac{x_{j}^{i}-x_{j-1}^{i}}{t_{j}^{i}-t_{j-1}^{i}}$	Average of the change in the current value from the previous, divided by the difference of follow up times. It is also known as average slope, and used in [27,31]

**Fig 1:**
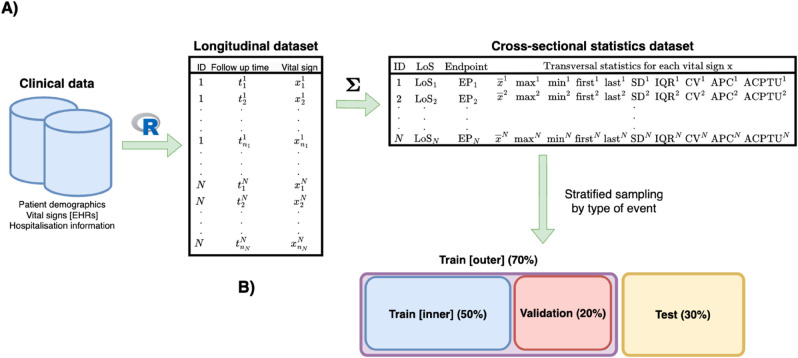
Vital signs - From longitudinal measurements to cross-sectional statistics. Top (A): Statistics calculated for vital sign x. Bottom (B): Data splitting for model evaluation.

Fig 1A illustrates the proposed workflow to calculate the cross-sectional statistics from a single longitudinal vital sign. This procedure was applied analogously for the rest of them, and stored in the same dataset. The use of cross-sectional statistics is motivated by the sparseness of the longitudinal vital signs and is a widely used methodology when modelling this type of data, as described in the introduction.

Fig 1B describes the random training-validation-test partition, proposed for modelling and evaluation.

In some episodes, certain vital signs were never measured (i.e. *n*_*i*_ = 0). Note that in this case, we assigned missing values to all cross-sectional statistics. Additionally, when only a single measurement was taken during the entire stay (i.e. *n*_*i*_ = 1), the variability-related statistics (SD, IQR, CV, APC, and ACPTU) were assigned zero values. Regarding the level of consciousness, we opted for collecting only the last observation. The discrete nature of this variable did not allow us to compute other statistics, such as mean or variance. Besides, in most episodes consciousness was reported at most only once, which does not generate true longitudinal data, so we described the behavior of this vital sign with just one statistic. The primary motivation for retaining this vital sign is its widespread use in clinically well-established in-hospital deterioration indices, such as the National Early Warning Score (NEWS) [37] and the Modified Early Warning Score (MEWS) [38].

**Fig 2:**
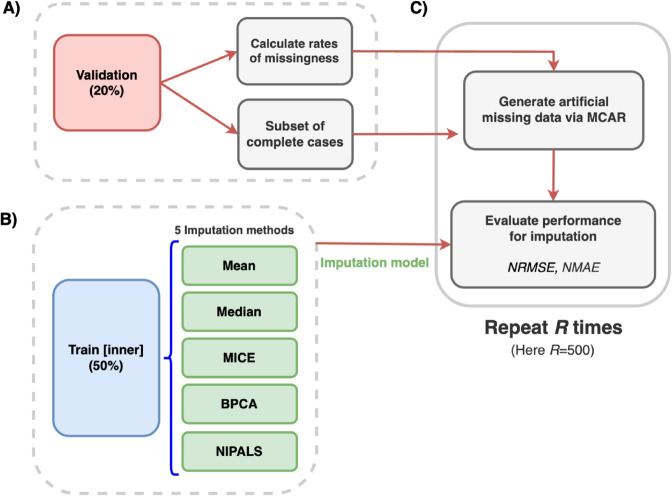
Methodology for evaluating missing data imputation strategies.

To cope with missing values in the cross-sectional dataset, we studied five different imputation strategies [39], and outlined in Fig 2. The following methods were evaluated: i) mean, ii) median, iii) multiple imputation by chained equations (MICE) with predictive mean matching (PMM) [40], iv) Bayesian principal components analysis (BPCA) [41], and v) non-linear estimation by iterative partial least squares (NIPALS) [42]. To determine the most suitable imputation strategy, we trained them on the ‘train [inner]’ subset (Fig 1B), and evaluated their performances on the validation subset. We accounted for both the normalised root mean squared error (NRMSE) and the normalised mean absolute error (NMAE), defined as follows:

$$\text{NRMSE}=\frac{\sqrt{𝔼[({\hat{y}}_{-}y{)}^{2}]}}{\text{SD}(y)},\;\text{NMAE}=\frac{𝔼[|\hat{y}-y|]}{\text{SD}(y)},$$ (1)

where $\hat{y}$ is the imputed value and *y* is the real groud-truth value (removed artificially - see Fig 2C).

Given that using cross-sectional statistics for vital signs entails generating a considerable number of covariates, potentially with noticeable inter-correlations, we deemed it necessary to perform a subsequent stage of variable selection. Exclusively for this variable selection stage, and following a Cause-Specific Cox (CSC) methodology [43], we estimated an auxiliary Cox Proportional Hazards (Cox-PH) model for episodes of one endpoint, treating episodes of the other endpoint as censored. This methodological approach is inherent to CSC and does not imply that the data are actually censored in the general sense; instead, episodes are artificially censored as already described, for the sole purpose of Cox-PH.

Then, Cox-PH was evaluated for variable selection using: i) Best Subset Selection (BeSS) [44], and ii) LASSO Regularised Cox Regression techniques [45]. Note that, whereas BeSS does not involve any hyperparameter choice, LASSO requires tuning its regularisation strength *λ*, which we did via 10-fold cross-validation.

During this procedure, for each strategy (either BeSS or LASSO), we generated two time-to-event regularised Cox-PH for variable selection, fitted on the ‘train [outer]’ (Fig 1B) set:

(a) One focused on the time-to-event for favourably discharged episodes, with deterioration episodes taken as censored data – yielding a selection set $s_{Fav}$; and
(b) another focused on deterioration episodes, with favourable discharges censored – which yielded *s*_*Det*_.

Thus, the final set of selected variables was the union of both $s=s_{Fav}\cup s_{Det}$.

### Competing risk models

As we considered two mutually exclusive endpoints, competing risk models (CR) are a suitable technique to study time-to-event outcomes, LoS here.

#### Linear models.

In CSC, hazard λ(·) reflects the instantaneous rate of occurrence for the *k*-th endpoint, in episodes that were currently endpoint-free. Let *D* be the type of endpoint. Then the cause-specific hazard function for endpoint *k*, given the covariate vector x is [46]:

$$\lambda_{k}^{CSC}(t|x)=\lim_{\Delta t\to 0}\frac{P\left(t\leq T<t+\Delta t,D=k|T\geq t,x\right)}{\Delta t},$$ (2)

where *T* is the random variable “time until the occurrence of the endpoint of interest”, t∈[0,∞), and in our scenario k∈{Favourable discharge, Deterioration}.

Under the sub-distribution hazard approach, also known as Fine and Gray (FG), the hazard λ(·) for endpoint *k* given the covariate vector x is [47]:

$$\lambda_{k}^{FG}(t|x)=\lim_{\Delta t\to 0}\frac{P\left(t\leq T<t+\Delta t,D=k|T>t\;\cup (T<t\cap D\neq k),x\right)}{\Delta t}.$$ (3)

The regression model that relates the hazard functions (either $\lambda_{k}^{CSC}$ or $\lambda_{k}^{FG}$) to a set of covariates, can be written as [43]:

$$\lambda_{k}(t|x)=\lambda_{0}(t)\exp\left(x\beta \right),$$ (4)

where $\lambda_{0}(t)$ denotes the baseline hazard function (i.e. the hazard function for a subject whose covariates are all set equal to zero) [43]. For FG model, $\lambda_{0}(t)$ is a completely unspecified, non-negative function in *t* [47].

Note that the risk sets in each model (2) and (3) differ: CSC considers only episodes currently endpoint-free, whereas FG incorporates also those that previously experienced a competing endpoint. Additionally, CSC estimates the effect of covariates on the cause-specific hazard function, whereas FG estimates their effect on the Cumulative Incidence Function (CIF) [43]. The ${\text{CIF}}_{k}(t)=P(T\leq t,D=k)$ reflects the probability of experiencing the *k*-th endpoint before time *t*, and before any different type of endpoint. The episode-specific CIF is denoted as $F_{k}(t|x)=P(T\leq t,D=k|x)$. Given an episode with covariate vector $x_{i}$, its empirical CIF is represented by ${\hat{F}}_{k}(t|x_{i})$ [46].

#### Ensemble learning.

Within the Random Forest ensemble learning paradigm, Random Survival Forests (RSF) have been proposed for analyzing competing risks [46]. In RSF, each of the *B* trees in the forest gets trained on a bootstrap sample from the original dataset. Building a tree consists in generating a hierarchy of meaningful decisions; from a root node, through intermediate branches, until terminal nodes (leaves). Splits are in the form: either z≤c or *z*>*c*, where *z* is an explanatory variable in x, and *c* is the splitting threshold. For each node, a total of M≤p candidates variables get considered, where *p* is the dimensionality of our covariate vector $x\in {\mathbb{R}}^{p}$, and typically M≪p to promote variance in the ensemble learning strategy.

In the context of CR, when judging a candidate variable *z* and a threshold *c*, two possible dissimilarity criteria are considered:

A. Generalised log-rank test (LR) – To reject the null hypothesis of equality of cause-specific hazards in the left (*l*) and right (*r*) sub-populations after the node split $H_{0,k}:\lambda_{k,l}^{CSC}(t)=\lambda_{k,r}^{CSC}(t)$.
B. Gray’s test (GT) – To reject the null hypothesis of equality of cumulative incidences functions $H_{0,k}:F_{k,l}(t)=F_{k,r}(t)$.

Consequently, at a certain branching node, the splitting variable *z* and threshold *c* are chosen by maximising greedily the specified dissimilarity criterion (LR or GT).

Each tree branch grows until the terminal condition is met: a leaf node should have no less than *n*_0_ unique cases. Hence, one often considers three main RSF hyperparameters: *B*, *M*, and *n*_0_. Here we fixed *B* = 100 trees, whereas we tuned *M* and *n*_0_ attending to out-of-bag (OOB) errors [46].

#### Model constraints and assumptions.

A complete understanding of CR dynamics requires careful analysis, including the application of two linear models: CSC and FG, along with recommendations for rigorous implementation [48]:

i. Using a distinct terminology for each model of the hazard ratio, for cause-specific Cox model and for Fine and Gray model.
ii. Reporting all the CSC and FG coefficients.
iii. Presenting the results in a unified interpretation to connect and reconcile results from the two sets of models.
iv. Checking the proportional hazard assumption, which ensures that the effect of covariates on the hazard is consistent over time, for both CSC and FG models.
v. Providing plots of cumulative incidences for all categorical variables.

In this article, the proportional hazards assumption, which ensures that the effect of covariates on the hazard remains consistent over time, was evaluated using Schoenfeld residuals [49]. In contrast, ensemble learning models do not require validation of this assumption.

### Baseline approaches

To establish meaningful performance baselines, we propose the following approaches:

A. To model LoS without covariates, applying each of the four CR models described above - Null baseline. For a given cause *k*, the hazard under the null model is:
  $$\lambda_{k}(t|x)=\lambda_{0}(t),$$ (5)
  which does not depend on covariates x. The baseline $\lambda_{0}(t)$ can be estimated using various approaches. For instance, it can be assumed constant, implying an underlying exponential distribution; alternatively, it can be estimated using parametric distributions or likelihood-based estimators [50].
B. To model LoS with sex, age, and one clinical EWS score:
  B.1. We opted for NEWS, as well as MEWS, given their widespread use within clinical contexts [51].
  B.2. We computed these scores using the last vital sign observation recorded within each episode.
  B.3. With those inputs, we used the four CR model described above.
  Calculating the NEWS score required a binary variable, ‘on supplemental oxygen (yes or no).’ This variable is not included in our proposed CR modelling. However, it will be described alongside other vital signs. For missing values of this variable, our clinical team established that a value of 0 should be assumed, disregarding any supplemental oxygen usage when no value was recorded.

### Performance in competing risks

Here we evaluated CR model performance attending to four magnitudes: i) Brier score (BS), ii) Integrated Brier score (IBS), iii) Concordance index (C-Index), and iv) Cumulative C-Index (CC-Index).

For an endpoint of type *k*, BS_*k*_ is the average squared difference between the true event status and the estimated risk [52]:

$${\text{BS}}_{k}(t)=𝔼\left[\{I(T<t,D=k)-{\hat{F}}_{k}(t|x){\}}^{2}\right],$$ (6)

where I(T<t,D=k) is the indicator function for event status, and ${\hat{F}}_{k}(t|x)$ is the estimated CIF.

Then, IBS corresponds to its integral along time:

$${\text{IBS}}_{k}(t)=\int_{0}^{t}{\text{BS}}_{k}(\tau )d\tau .$$ (7)

On the other hand, for an endpoint of type *k*, C-index estimates the probability that, given a random pair of episodes, the one that experienced the event first has a higher predicted outcome than the other episode [53]:

$${\text{C}}_{k}(t)=P\left(F_{k}(t|x)>F_{k}(t|x)\;|\left(D_{i}=k\;\cap \;\left(\;T_{i}<T_{j}\;\cup \;D_{j}\neq k\right)\right)\right),\;j=1,\ldots ,n,$$ (8)

where *T*_*i*_ and *T*_*j*_ are the times-to-endpoint for episodes i≠j.

Since we computed the C-Index at m∈ℕ discrete evaluation times $\left\{t_{1},t_{2},\ldots ,t_{m}\right\}$, here we defined an ad hoc cumulative CC-Index:

$${\text{CC}}_{k}(m)=\sum_{i=1}^{m}{\text{C}}_{k}(t_{i}).$$ (9)

It is important to exercise caution when using the CC-Index, as it has limitations. This metric is only applicable when comparing the performance of models evaluated at identical time points. To compare models across different evaluation periods, an alternative approach may be more appropriate. One such option is the average C-Index, where the CC-Index is used as the numerator and constant *m* serves as the denominator.

In this work, we evaluate model performance over a time frame of 14 days for all models (i.e. *m* = 14). The specific lengths of stay range from 2 to 15 days of hospitalisation.

## Results

### Study cohort

A total of 22,512 episodes were recorded, among which N=19,602 (87.07%) met the inclusion criteria. According to our clinical definitions, 18,750 episodes (95.65%) were favourably discharged, whereas 852 (4.35%) experienced deterioration. Table 2 describes the main characteristics of our cohort.

**Table 2:** Descriptive of our cohort. For categorical variables: counts of cases (percentage). For numerical: median ($25^{\text{th}}$ – $75^{\text{th}}$ percentiles).

Variable/Categories		Total episodes	Favourable discharge	Deterioration		
		*N*=19,602	*N*_*F*_=18,750 (95.65%)	*N*_*D*_=852 (4.35%)	*p-value	**Effect size
Sex	Male	10,940 (55.81%)	10,433 (55.64%)	507 (59.51%)	0.028	0.01 – Tiny
	Female	8,662 (44.19%)	8,317 (44.36%)	345 (40.49%)		
Age [years]		70.06 (55.80 – 81.10)	69.62 (55.26 – 80.64)	79.03 (69.03 – 86.82)	≪ 0.001	0.35 – Large
Body temperature [^°^C] (Mean in episode)		36.57 (36.37 – 36.75)	36.57 (36.37 – 36.75)	36.70 (36.42 – 37.03)	≪ 0.001	0.23 – Medium
Systolic BP [mmHg] (Mean in episode)		125.67 (114.40 – 138.50)	126.00 (114.78 – 138.75)	118.17 (105.83 – 131.64)	≪ 0.001	0.24 – Medium
Diastolic BP [mmHg] (Mean in episode)		72.18 (67.00 – 77.78)	72.29 (67.10 – 77.80)	69.33 (63.03 – 76.65)	≪ 0.001	0.17 – Small
Heart rate [beats/min] (Mean in episode)		75.00 (66.73 – 84.12)	74.60 (66.50 – 83.45)	88.17 (77.67 – 98.14)	≪ 0.001	0.47 – Very large
Respiratory rate [breaths/min] (Mean in episode)		18.00 (16.00 – 20.00)	18.00 (16.00 – 19.98)	20.00 (17.87 – 23.00)	≪ 0.001	0.36 – Large
Oxygen saturation SpO_2_ [%] (Mean in episode)		96.00 (94.82 – 97.23)	96.10 (94.96 – 97.25)	94.50 (92.54 – 96.00)	≪ 0.001	0.45 – Very large
Level of consciousness (Last in episode)	Level I	6,932 (35.36%)	6,752 (36.01%)	180 (21.13%)	≪ 0.001	0.24 – Medium
	Level II	208 (1.06%)	145 (0.77%)	63 (7.39%)		
	Level III	67 (0.34)%	18 (0.10%)	49 (5.75%)		
	Missing	12,395 (63.24%)	11,835 (63.12%)	560 (65.73%)		
Supplementary oxygen	Yes	2,915 (14.87%)	2,400 (12.80%)	515 (60.44%)	≪ 0.001	0.27 – Medium
	No	14,409 (73.51%)	14,151 (75.47%)	258 (30.28%)		
	Missing	2,278 (11.62%)	2,199 (11.73%)	79 (9.28%)		
LoS [days]		3.82 (1.98 – 6.66)	3.76 (1.97 – 6.38)	6.66 (3.24 – 12.65)	≪ 0.001	0.34 – Large

*p-value for Mann–Whitney U test for numerical variables, Chi-square test for categorical.

**Rank-biserial correlation for numerical variables, Cramer’s *V* for categorical. Interpretation as in [54].

**Fig 3:**
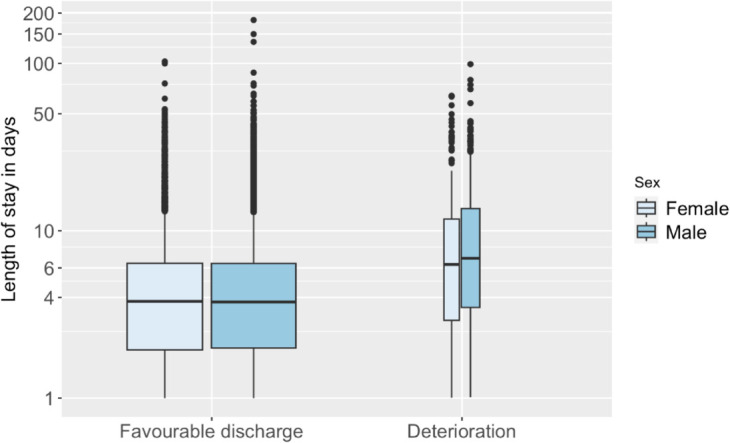
Distribution of LoS, by endpoint and by sex. Note that the *Y*-axis is on a logarithmic scale, and that the widths of the boxplots reflect the discrepancy in the number of episodes for each endpoint.

The distribution of LoS, by endpoint, and by sex, is visualised in Fig 3. For favorable discharges, the median LoS ($25^{\text{th}}$ – $75^{\text{th}}$ percentiles) was 3.78 (1.94 - 6.39) days for females; for males, the median was 3.75 (1.99 - 6.37) days. For deterioration, the median LoS was 6.29 (2.91 - 11.75) days for females; for males, the median was 6.84 (3.49 - 13.49) days.

We conducted Mann-Whitney U tests to evaluate differences in LoS between males and females for both favorable discharge and deterioration outcomes. For favorable discharge, the test revealed no significant difference in LoS between males and females (W=43,827,607; *p* = 0.23), with an effect size *r* = 0.01, indicating a tiny effect [54]. For deterioration, the test showed a significant difference in LoS between the sexes (W=94,406; *p* = 0.048), with an effect size *r* = 0.01, which also reflects a tiny effect.

### Imputation and variable selection

Table 3 reflects the missing rates before and after converting longitudinal data into cross-sectional statistics. Even though each vital sign generates 10 different statistics, their missing rate is constant across those ten (i.e. within each vital sign), as reported in Table 3. As can be seen, even after cross-sectional transformation, the respiratory rate continues to have a high rate of missing data.

**Table 3:** Missing rates for vital signs.

Vital sign	Longitudinal	Cross-sectional
Body temperature	30.82%	0.07%
Systolic BP	44.55%	0.04%
Diastolic BP	44.50%	0.04%
Heart rate	43.40%	0.04%
Respiratory rate	96.31%	87.01%
Oxygen saturation SpO_2_	55.38%	5.45%
Level of consciousness	92.91%	63.24%

Table 4 summarises the performances – in terms of NRMSE and NMAE – by each imputation strategy. MICE outperformed all other techniques, both in NRMSE and NMAE.

**Table 4:** Performance by each imputation method, NRMSE↓ and NMAE↓ – Mean and standard deviation (SD). In bold, the best performances, i.e. the lowest errors.

Method	NRMSE↓	NMAE↓
	Mean ± SD	Mean ± SD
*Mean*	0.172 ± 0.329	0.100 ± 0.227
*Median*	0.175 ± 0.335	0.099 ± 0.224
*MICE*	0.138 ± 0.290	0.083 ± 0.192
*BPCA*	3.886 ± 1.532	2.832 ± 1.095
*NIPALS*	0.420 ± 0.233	0.306 ± 0.171

**Fig 4:**
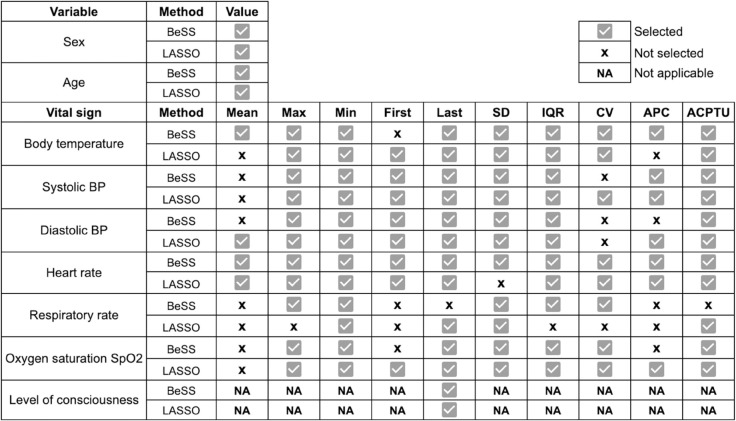
Results for variable selection – BeSS vs LASSO.

Variable selection results are detailed in Fig 4. Most of the statistics for respiratory rate were discarded. Whereas the minimum was never excluded, the mean was discarded in the majority of vital signs. The level of agreement between BeSS and LASSO is moderate. Generally, they did not discard the same statistics across vital signs. In terms of the number of discards, BeSS and LASSO exhibit high similarity, with BeSS discarding a total of 14 statistics whereas LASSO discarded 12.

### Competing risk modelling

#### Performance.

Table 5 shows the different performances, in terms of IBS. It illustrates that models employing the full set of variables exhibit a superior performance for favourable discharge. For deterioration, FG and GT-RSF models showed the highest performance with the full set, whereas CSC performed best with BeSS, and LR-RSF prevailed with LASSO.

**Table 5:** IBS↓[×100]. In bold, the set-up with the highest overall performance by endpoint. In italics, the best scenario for each type of CR model.

	Favourable discharge				Deterioration			
	CSC	FG	LR-RSF	GT-RSF	CSC	FG	LR-RSF	GT-RSF
*Null*	16.394	16.394	16.394	16.394	2.199	2.199	2.199	2.199
*NEWS*	15.753	15.793	15.726	15.723	1.964	1.964	1.979	1.997
*MEWS*	15.532	15.54	19.305	15.549	1.911	1.919	1.909	1.935
*BeSS*	8.018	8.966	9.549	9.319	*1.709*	1.750	1.902	1.902
*LASSO*	8.010	8.963	9.590	9.356	1.711	1.749	*1.588*	1.896
*Full*	*7.996*	*8.946*	*2.086*	**2.069**	1.714	*1.731*	2.164	**0.386**

**Fig 5:**
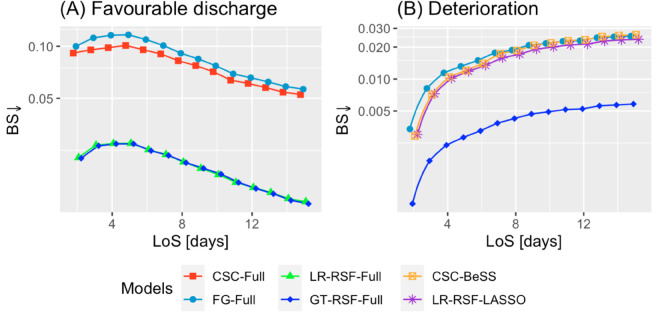
BS↓ performance for the best case for each type of CR model. *Y*-axis in logarithmic scale.

Fig 5 illustrates the BS results for those pairs CR model - variable set highlighted in Table 5. For favourable discharge (Fig 5A, GT-RSF-Full and LR-RSF-Full performed the best, with BS below 0.04 throughout the entire time window. On the other hand, Fig 5B shows that the best set-up for deterioration was GT-RSF-Full, with BS values below 0.006.

The CC-Index over the time window is shown in Table 6. It illustrates that favourable discharge exhibits superior performance when models use the full set of variables, except in CSC. For deterioration, ensemble models showed the highest performance with the full set of variables, whereas CSC model performed best with LASSO, and FG prevailed with BeSS.

**Table 6:** CC-Index↑. In bold, the set-up with the highest overall performance by endpoint. In italics, the best scenario for each type of CR model.

	Favourable discharge				Deterioration			
	CSC	FG	LR-RSF	GT-RSF	CSC	FG	LR-RSF	GT-RSF
*Null*	7.000	7.000	7.000	7.000	7.000	7.000	7.000	7.000
*NEWS*	8.299	8.295	8.256	8.234	11.975	11.984	11.867	11.863
*MEWS*	8.328	8.331	7.586	8.277	11.759	11.784	11.837	11.803
*BeSS*	*11.613*	11.857	11.304	11.519	12.422	*12.374*	12.408	11.652
*LASSO*	11.604	11.850	11.507	11.581	*12.423*	12.349	12.408	11.652
*Full*	11.611	**11.864**	*11.640*	*11.674*	12.369	12.343	*12.475*	**13.929**

**Fig 6:**
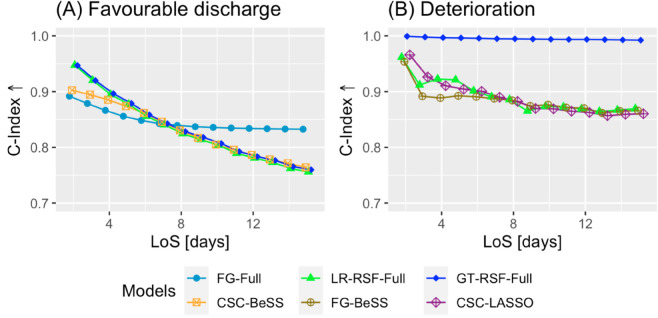
C-Index↑ performance for the best case for each type of CR model.

Fig 6 illustrates the C-Index results for the pairs of CR model and variable set highlighted in Table 6. Fig 6A shows that models GT-RSF-Full and LR-RSF-Full achieved the best performance for favourable discharge, from day 2 until day 7. However, from day 8 onwards, the FG-Full model demonstrated the highest performance. On the other hand, Fig 6B shows that, for deterioration, the best performance was achieved by the GT-RSF-Full model.

Another widely used performance metric is the time-dependent Area Under the Receiver Operating Characteristic Curve (time-dep AUC) [55]. Following the same approach as with BS and C-Index, time-dep AUC results are presented in the Supplementary Materials (S8 Fig), allowing further confirmation of the effectiveness of our modeling approach.

#### Ensemble learning – Findings.

Each ensemble model was subjected to a comprehensive tuning process aimed at identifying the optimal hyperparameters. The specific hyperparameter values selected for each model, along with the detailed configuration settings, are reported in the supplementary materials, Appendix C, Table D.

**Fig 7:**
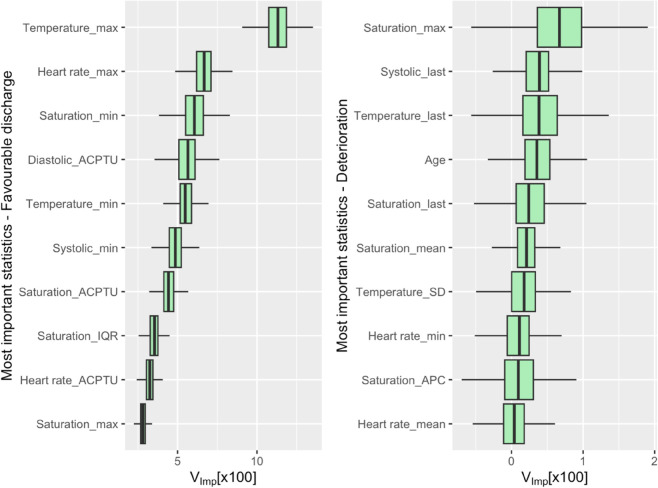
Boxplot of the top ten highest V_Imp_[×100] for GT-RSF-Full. Left: Favourable discharge, right: Deterioration.

To evaluate the contribution by each covariate in explaining LoS, we computed variable importance (V_Imp_) using the method proposed by [56], which involves random permutations of variables and out-of-bag (OOB) performance. We applied this method with the best model available, i.e. GT-RSF-Full, allowing us to rank the covariates according to V_Imp_ Fig 7 depicts the top ten magnitudes for favourable endpoint in LoS (left), and deterioration (right). The cross-sectional statistics with the highest importance were ‘max’ and ‘last’ for deterioration, and ‘max’ for favourable discharge. Among the vital signs, body temperature, and saturation frequently appeared in the top-ten V_Imp_.

V_Imp_ was also calculated for LR–RSF-Full (see supplementary materials: Appendix S.B, S1 Fig). For this model, the cross-sectional statistics with the highest importance were ’max’ and ’min’ for both deterioration and favorable discharge. Regarding vital signs, body temperature had the highest importance for favorable discharge, followed by diastolic BP. For deterioration, the most important vital signs were diastolic BP, followed by temperature. The most frequently appearing vital signs in the top ten V_Imp_ were saturation and temperature for favorable discharge and deterioration, respectively.

The similarities between V_Imp_ for GT-RSF-Full and LR-RSF-Full reveal useful insights. For favorable discharge, the maximum body temperature emerges as the most important covariate, showing a notable difference with respect to the others. Additionally, nine out of the ten most important covariates for favorable discharge remain consistent between the two models, although their order differs. For deterioration, three of the ten most important covariates are shared between GT-RSF-Full and LR-RSF-Full, with the maximum value being the most important statistic in both.

Conversely, there are some dissimilarities between the two models. In GT-RSF-Full for favorable discharge, the ACPTU of heart rate is included in the top ten covariates, whereas it is absent in LR-RSF-Full. Instead, the CV of SpO_2_ appears in LR-RSF-Full’s top ten. For deterioration, only the minimum heart rate, maximum saturation, and the last value of systolic BP remain among the top ten covariates, indicating that all other covariates differ between the two models.

#### Linear models – Findings.

We chose to describe CSC-Full and FG-Full models, which demonstrated good overall performance and included the full set of variables. The recommendations outlined by [48] were considered and applied in this article.

The estimated coefficients for CSC-Full and FG-Full are reported in the supplementary materials. Table B in Appendix S.B reflects the estimated coefficients for CSC. As shown, the largest coefficients (in absolute value) were related to CV statistic. For favourable discharge, the largest coefficients (in absolute value) are related to CV of temperature and saturation, whereas for deterioration, they are associated with the CV of temperature and heart rate. However, for favourable discharge, the CV of temperature was high but not significant. As shown in Table C of Appendix S.B, the FG model also indicates that the largest coefficients (in absolute value) are associated with the CV statistic. For favourable discharge, the highest coefficient is related to the CV of saturation, whereas for deterioration, it is the CV of temperature. Other vital signs with significant coefficients for favourable discharge include consciousness level III, saturation, and systolic BP. For deterioration, heart rate and systolic BP exhibit the largest estimated coefficients.

Following the methodology outlined by [49], we evaluated the proportional hazard assumption by plotting Schoenfeld residuals against LoS for each covariate. According to this assumption, the residuals should exhibit a constant mean over time. To assess this, we included scatterplot smoothers for each covariate. All residual plots are provided in the supplementary materials: Appendix S.B, S2 Fig for CSC-Full related to favourable discharge; Appendix S.B, S3 Fig for FG-Full related to favourable discharge; Appendix S.B, S4 Fig for CSC-Full related to deterioration; and Appendix S.B, S5 Fig for FG-Full related to deterioration.

For favourable discharge, both the CSC and FG models exhibit some similarities: IQR shows a non-linear trend across all vital signs; consciousness levels II, III, and Missing (conscious_nm) also display non-linear effects; additionally, both sex and age variables present non-linear effects in both models. However, there are some differences: in FG-Full model, ACPTU shows a non-linear trend across all vital signs, whereas it does not in the CSC-Full model; moreover, most statistics related to respiratory and heart rates also display non-linear trends in FG-Full. Overall, the FG-Full model reveals more non-linear trends compared to CSC-Full.

For deterioration, both the CSC and FG models show similarities to those observed for favourable discharge: non-linear trend for IQR, consciousness levels II, III and Missing, sex and age variables present non-linear effects in both models. However, there are differences: in FG-Full model, ACPTU shows a non-linear trend across all vital signs, whereas it does not in the CSC-Full model; most statistics related to systolic and diastolic BP display non-linear trends in FG-Full. As for favourable discharge, the FG-Full model reveals more non-linear trends compared to CSC-Full.

Finally, estimated CIF for sex and consciousness level (all categorical variables) are depicted in supplementary materials: Appendix S.B, S6 Fig for sex and Appendix S.B, S7 Fig for consciousness level. The CIFs are estimated using the Aalen-Johansen estimator [57].

## Discussion

### Main findings

In this study, we proposed a workflow to model the LoS of hospitalisation episodes at the GUUH, using sex, age, and cross-sectional statistics of vital signs as covariates for linear and ensemble CR models. We gathered EHR data from a mid-sized hospital for the year 2019. We selected this year to avoid the profound alterations in clinical management caused by the COVID-19 pandemic.

Our proposal presents a comprehensive, rigorous, and straightforward approach to modelling LoS, including data pre-processing and time-to-event CR models. The data pre-processing addressed the management of sparse measurements taken at irregular time points, aiming to provide a clinically understandable transformation of data by summarising the longitudinal vital signs into a set of cross-sectional statistics. This process also involved a thorough comprehensive evaluation of methods to cope with missing data, using five distinct approaches: mean, median, MICE, BPCA, and NIPALS. We use NRMSE and NMAE to determine which imputation strategy worked best here. Furthermore, we applied two well-known variable selection algorithms: BeSS and LASSO to determine which covariates were informative for LoS modelling and which were redundant, and therefore candidates for being discarded.

For time-to-event modelling, we used CR models. Two linear ones: CSC and FG, as well as one ensemble learning RSF-method. For RSF, two alternative splitting rules were explored: LR (to maximize the difference in cause-specific hazards) and GT (to maximize the difference in CIFs). Furthermore, a comprehensive set of four performance metrics was used to evaluate which CR set-up yields the best results: BS, ISB, C-Index, and CC-Index. Lastly, using (V_Imp_) we quantified the importance of the demographic information and the vital signs statistics, to aid in the clinical interpretation of our best-performing method.

Our findings show that GT-RSF-Full notably outperformed all others. An interesting agreement was observed between the CC-Index results and the IBS, confirming its superiority over the other proposals. In general, the most informative statistics for this model were maximum and last values, whereas the most important vital signs for this model were oxygen saturation SpO_2_ and body temperature.

Our analysis revealed that SpO_2_ emerged as one of the most prominent vital signs. For each endpoint, 40% of the top-ranked variables were derived from SpO_2_, representing the highest proportion attributed to any single vital sign. However, SpO_2_ is the third most frequently missing vital sign in both longitudinal and cross-sectional formats (see Table 3), with a missing rate of 5.45% in the cross-sectional data. While this missing rate is relatively low and manageable, especially in clinical datasets, we still addressed it using Multiple Imputation by Chained Equation (MICE). Given the evidence that casewise deletion can lead to severely biased and imprecise regression coefficients estimates [58,59], we believe that imputing missing values, rather than excluding incomplete cases, provides more accurate estimates. Furthermore, MICE has been demonstrated to be effective in handling missing clinical data [60], making it a preferable approach to restricting our analyses to complete cases. Moreover, the Random Survival Forest (RSF) model consistently ranked aggregated forms of SpO_2_ among the most important features for both discharge and deterioration outcomes. This suggests that despite the missingness, when SpO_2_ data is available, it holds significant predictive value in the RSF model.

Besides, GT-RSF-Full clearly outperforms those baselines routinely available on clinical information (i.e. NEWS and MEWS scores) using the same CR techniques. This indicates that the vital signs and pre-processing methods we suggested (conversion to cross-sectional statistics and imputation) are capable of providing relevant information to better characterise LoS.

The findings of our study highlight the crucial role of SpO_2_ and body temperature as the most important predictors of patient deterioration. Previous research is in alignment with our results: statistically significant differences in vital signs, including SpO_2_, heart rate, respiration rate and systolic BP, have been reported between stable and unstable encounters, emphasizing the necessity for careful monitoring in clinical settings [61]. Furthermore, the maximum and ACPTU values of temperature, along with the minimum and ACPTU values of SpO_2_, within 24 hours prior to a deterioration event, were identified as significant indicators of clinical instability [25].

Moreover, the evidence indicates that trends in vital signs can provide more accurate predictions than isolated measurements. For instance, the area under the ROC curve for the trend values of minimum SpO_2_ was found to be superior to its last values in [27]. Additionally, the deterioration of SpO_2_ was frequently linked to higher 30-day mortality rates, particularly in patients with chronic respiratory conditions, emphasizing its role as a vital indicator of patient fragility [62]. This reinforces the need for integrating continuous monitoring of SpO_2_ and body temperature into clinical practice to identify at-risk patients and intervene proactively, ultimately improving patient outcomes as seen in [31].

The integration of CR models into healthcare practice has the potential to enhance the management of patients’ LoS in hospitals. This should enable healthcare providers to make more informed decisions regarding the allocation of resources, discharge planning and patient flow management. The accurate modelling of LoS can facilitate the prioritisation of patients for early discharge or transfer to lower-acuity settings, thereby optimising bed utilisation and reducing overcrowding. Furthermore, the understanding of LoS through CR models can assist in identifying patients at risk of prolonged hospitalisation, enabling targeted interventions to minimise complications and expedite recovery. This integration should ultimately support more efficient healthcare delivery, improved patient outcomes and enhanced operational efficiency in hospital settings.

Incorporating vital sign cross-sectional statistics as covariates in our modelling approach merits consideration. By summarising longitudinal vital signs into statistical descriptors, we can gain potentially valuable insights into each episode’s main behavior and its impact on LoS. Our finding that cross-sectional statistics enriches modelling is consistent with findings from other studies: for example, [27] found that incorporating vital signs trends significantly increased the accuracy of models designed to detect critical illness on the wards. Similarly, [29] observed that summary statistics (such as minimum, maximum, first, and last values) improved the real-time prediction of mortality for ICU patients compared to well-established clinical scores. Besides, [31] demonstrated that employing summary statistics and appropriate imputation methods can enhance model discrimination and reduce bias, although the clinical relevance of these improvements remains uncertain. Notwithstanding these studies, challenges such as data pre-processing and the selection of appropriate statistics necessitate careful consideration and further validation.

Data missingness is a major issue when working in clinical scenarios [60,63]. Most studies on LoS modelling consider only complete-case analyses: i.e. episodes with missing data are excluded. Other works use mean-value [34,64] or median-value imputation [6,16]. Another frequent imputation method is Last Observation Carried Forward (LOCF) [65,66], which tends to be preferred by clinicians, due to its simplicity and practicality in real-world healthcare settings. However, none of these studies compared different imputation methods systematically. Thus, we deemed it imperative to conduct a comprehensive comparison of various methods for statistical rigor: including multivariate techniques (MICE, BPCA, and NIPALS), which account for the interrelationships between variables based on the observed data patterns. Whereas these multivariate methods may pose challenges in clinical practice (compared to simpler techniques like mean and/or median imputation), our findings indicate that MICE in particular yielded a superior performance. Additionally, in numerous scenarios, approaches like LOCF and mean or median imputation can lead to biased estimates of statistics, such as regression coefficients [60].

We used NRMSE and NMAE to evaluate the performance of imputation, using complete information. To assess its effectiveness, it is crucial to evaluate the performance of MICE in comparison to the complete dataset with full observations [43]. This evaluation allows researchers to gauge the accuracy of imputed values and understand the impact of missing data on the overall analysis. By comparing the results derived from imputed data to those obtained from the full dataset, MICE can be validated as an effective method for minimizing bias and improving the reliability of statistical estimates.

Variable selection may be an effective tool for improving modelling in medical tasks, aiding in reducing the dimensionality of data, and in decreasing issues like redundancy and collinearity [67]. Here, we employed two methods: BeSS and LASSO Regularized Cox Regression. However, ensemble RSF methods considering the full set of variables outperformed the linear models. Superiority may be attributed to their capability to handle high-dimensional data [46], thus addressing the curse of dimensionality.

The application of variable selection methods must be approached with caution. This process might result in the exclusion of clinically meaningful variables, leading to a potential loss of crucial information and/or model underfitting. In turn, this might result in a reduction in performance of the CR models. Besides, the choice of selection method may introduce bias; for instance, LASSO tends to shrink coefficients towards zero, potentially omitting variables with small but meaningful effects. Similarly, BeSS can be computationally intensive and prone to overfitting if not carefully managed. Therefore, it is essential to evaluate the trade-off between the benefits and drawbacks of variable selection, examining the performances attained by each strategy and ensuring the clinical utility of the models.

Yet, linear models outperformed the three proposed baselines. Nonetheless, ensemble methods outperformed these linear models, particularly when predicting deterioration as the endpoint of interest. This superiority is likely attributable to the RSF’s ability to capture non-linear dependencies and complex relationships [46]. In any case, linear models are computationally more efficient than RSF, they do not require hyper-parameter tuning (except for variable selection purposes), and they are intrinsically explainable for humans by design (Beta coefficients). Consequently, although ensemble models showed the best performance, linear models might also be worth considering for modelling LoS, given these advantages.

Using linear models requires careful consideration, particularly in relation to their underlying assumptions. In our case, we assessed the proportional hazards assumption using a graphical test of residuals. Whereas not all variables fully met this assumption, we opted to use these models because they still offer valuable insights. The complexity of our dataset makes it difficult to perfectly satisfy every assumption. Nevertheless, given the models’ reasonable performance and to ensure a comprehensive exploration of methods for modelling LoS, we chose to include both the CSC and FG models in our analysis.

Performances in CR are typically reported using BS or IBS (sometimes as well) the C-Index, but rarely together. We employed these indicators simultaneously, alongside an ad hoc cumulative version of the C-Index, which we termed the CC-Index. This latter was aimed at aggregating C-index values through in time window of interest into a single indicator, offering a simplified means for comparison. We conducted a comprehensive examination regarding the combination between subsets of selected variables on the one side, and CR modelling techniques on the other; attending to the various performance metrics explained above. The IBS metric consistently demonstrated the superiority of GT-RSF-Full over all other models. For deterioration, the difference in performance by this model was remarkable with respect to the rest. In contrast, for favorable discharge, GT-RSF-Full yielded results similar to those for LR-RSF-Full, both models being noticeably superior over the remaining.

On the other hand, the CC-Index agreed in identifying GT-RSF-Full as the best method for modelling deterioration. However, for favorable discharge, it was FG-Full that performed the best according to the CC-Index, though the difference in performance was marginal compared to rest. This indicates that, whereas GT-RSF-Full for deterioration was consistently strong across most metrics and outcomes; the choice of the most suitable model for favorable discharge may vary depending on the performance metric used (with FG-Full showing a slight advantage according to the CC-Index).

### Limitations and future research

Several limitations may impact our findings. First, concerning the transformation from longitudinal measurements into cross-sectional statistics: The extraction of these statistics may result in a loss of information, encapsulated within the temporal trends and present in the original dataset. Simplifying longitudinal vital signs into cross-sectional statistics can result in the loss of temporal granularity, making it difficult to detect subtle dynamic patterns such as anomalies or abrupt shifts, which may be critical to building accurate models. As detailed in [68], cross-sectional statistics are often selected manually by a researcher for a given dataset. However, it is not guaranteed that those statistics chosen will necessarily be optimal for further analysis. In our study, we selected statistics aimed to facilitate the clinical understanding of longitudinal transformations. Specifically, we chose a set of ‘easy-to-interpret’ statistics that are meaningful from a clinical perspective. The main reason for this choice was to address the high rates of missing data and the temporal irregularity in the measurement.

Second, using the respiratory rate as a covariate was challenging, given its high missing rates. Some authors like [27,69] remark on the importance of respiratory rate in the early detection of deterioration. This motivated us not to discard respiratory rate data, even with the few that we had available in our dataset observations.

Third, employing MICE as an imputation method for missing data in cross-sectional statistics. As previously noted, MICE reduces bias in modelling by leveraging the relationships between variables, thereby improving the performance of imputation. However, it is important to acknowledge some limitations. MICE lacks a clear theoretical foundation [70]: while MICE relies on an iterative process akin to a Monte Carlo Markov Chain, its general properties are not rigorously proven. Then, the justification for MICE’s effectiveness has been based on empirical studies rather than strong theoretical arguments [71]. The lack of theoretical foundation could lead to over-fitting or under-fitting issues in the model, compromising the generalisability of the model when applied to new datasets or different populations. However, we used a ‘train-validation-test’ approach and a complete case evaluation, to reduce these issues.

Another limitation of MICE is non-convergence, which occurs when algorithm fails to reach a stable solution after several iterations [70]. If MICE does not converge, the imputation process becomes unreliable, and the results may fluctuate leading to incorrect conclusions. Additionally, a limitation arises when datasets contain many variables, making it difficult to determine which ones to include in the imputation process. Including too many variables can result in a complex and unstable model, a common issue in modelling, not exclusive to MICE. Large and complex models may slow down the imputation process or even cause it to fail. Simplifying the model without losing important information is crucial, but often challenging in practice.

Fourth, this was a single-center study and the results may not apply to other hospitals, with different populations and/or clinical routines. Whereas the patient population and clinical practices at our hospital may differ from those at other institutions (e.g. the definition of consciousness levels), we believe that our findings could still be relevant to similar healthcare environments, particularly those with comparable clinical protocols. However, further research (including multi-center studies and studies in different healthcare systems) is necessary to validate the broader applicability of our results.

We plan to orient our forthcoming efforts on evaluating longitudinal imputation methods for sparse clinical data and exploring the application of models designed for longitudinal repeated measurements in time-to-event outcomes. Specifically, we aim to investigate how these methods can enhance the modelling for time-to-event outcomes, such as LoS. Additionally, we will assess the integration of more comprehensive clinical information, including laboratory results and comorbidities, to improve the robustness and predictive power of these models.

## Conclusions

We applied time-to-event CR to model in-hospital LoS, considering two mutually exclusive endpoints. By transforming longitudinal vital signs into cross-sectional statistics, we notably reduced data missingness. Our pre-processing involved a thorough exploration of imputation techniques and variable selection methods. Subsequently, we studied both linear CR models and RSF ensembles. RSF turned out to consistently outperform linear models. Furthermore, examining variable importance may help provide clinicians with practical insights.

## Supporting information

S1 File On-line supplementary materials. (S.A) Data Pre-processing: Feasible ranges for vital signs. (S.B) Competing Risks Modeling: Outputs from the competing risks models. (S.C) Hyperparameters: Detailed specifications for each ensemble learning model. The R code used for data pre-processing, baseline models, and final modeling is available in the associated GitHub repository https://github.com/jc-espinosa/Article_LoS.
(PDF)
