# Supplementary material for: Modelling in-hospital length of stay: A comparison of linear and ensemble models for competing risk analysis
Source: PLoS One. 2025 Aug 26;20(8):e0322101. doi: 10.1371/journal.pone.0322101 (PMC12380278; doi:10.1371/journal.pone.0322101)
Supplement: S1 File — (S.A) Data Pre-processing: Feasible ranges for vital signs. (S.B) Competing Risks Modeling: Outputs from the competing risks models. (S.C) Hyperparameters: Detailed specifications for each ensemble learning model. The R code used for data pre-processing, baseline models, and final modeling is available in the associated GitHub repository https://github.com/jc-espinosa/Article_LoS. (PDF) [file pone.0322101.s001.pdf]

On-line Supplementary Materials

S.A. Data pre-processing

Table A: Feasible ranges for each vital sign.

Vital sign	Unit	Minimum	Maximum
Body temperature	[ºC]	30	42
Systolic BP	[mmHg]	40	280
Diastolic BP	[mmHg]	20	150
Heart rate	[beats/min]	20	250
Respiratory rate	[breaths/min]	8	50
Oxygen saturation SpO_2	[%]	60	100

S.B. Competing Risks Modelling

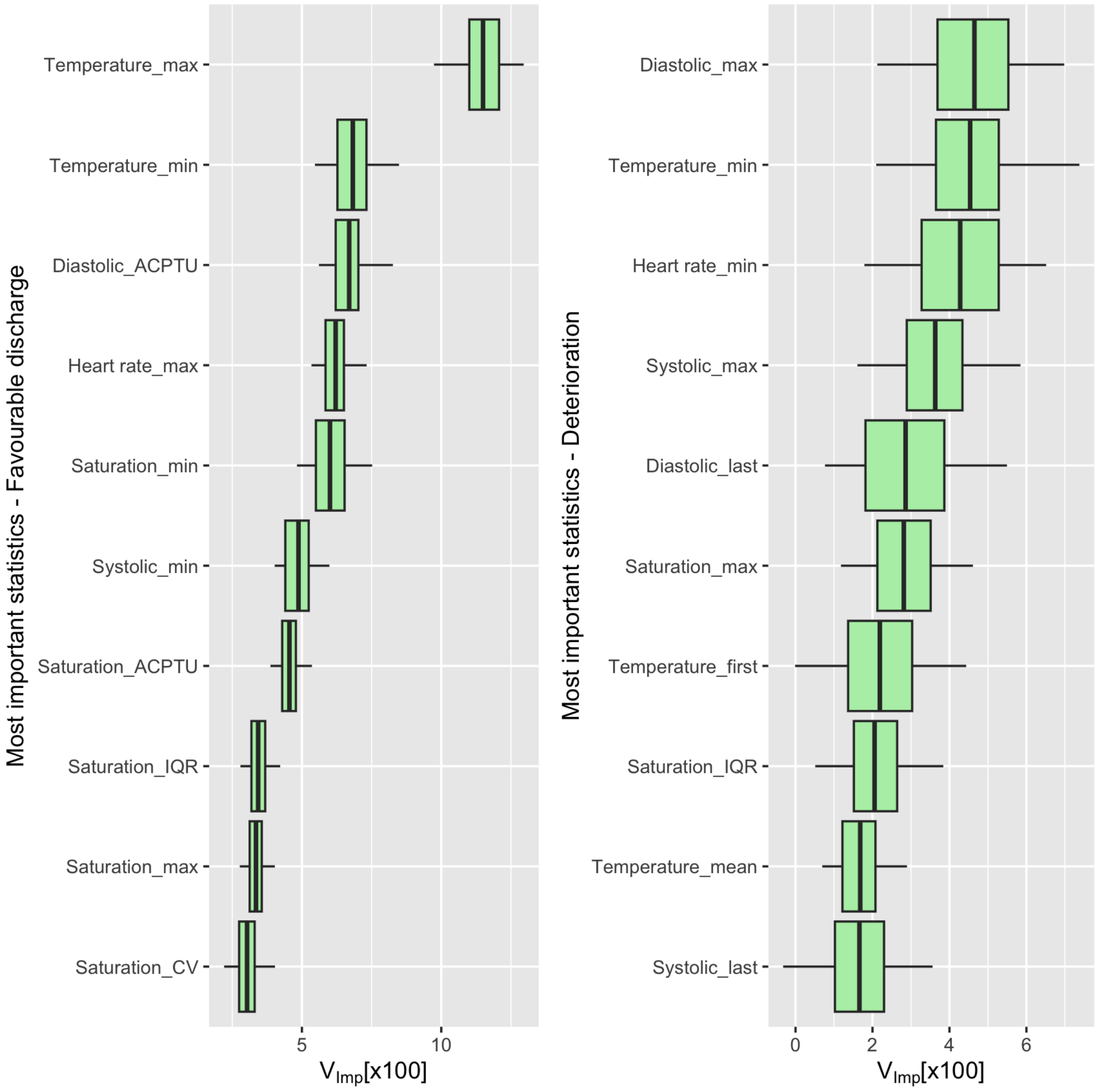

Fig S1. Error-plot of the top ten highest $V_{IMP}[\times 100]$ for LR-RSF-Full. Left: Favourable discharge, right: Deterioration.

Table B: CSC-Full estimated coefficients. Significance codes: *** for p-value < 0.01; ** for p-value < 0.05; * for p-value < 0.1.

Covariate	Favourable discharge			Deterioration		
	$\hat{\beta}$	s.e	p-value	$\hat{\beta}$	s.e	p-value
Temperature_max	-1.41e+00	4.97e-02	$\ll$ 1e-03 ***	-7.18e-01	1.47e-01	$\ll$ 1e-03 ***
Systolic_max	-2.14e-02	2.00e-03	$\ll$ 1e-03 ***	-3.46e-02	7.90e-03	$\ll$ 1e-03 ***
Diastolic_max	-4.73e-02	3.29e-03	$\ll$ 1e-03 ***	-1.21e-02	1.26e-02	3.37e-01
Resp rate_max	1.52e-02	1.64e-02	3.55e-01	-4.94e-03	6.08e-02	9.35e-01
Heart rate_max	-2.28e-02	2.12e-03	$\ll$ 1e-03 ***	-4.50e-02	7.31e-03	$\ll$ 1e-03 ***
Saturation_max	-1.14e-01	1.56e-02	$\ll$ 1e-03 ***	-3.86e-02	4.94e-02	4.34e-01
Temperature_min	1.83e+00	5.92e-02	$\ll$ 1e-03 ***	7.44e-01	1.77e-01	$\ll$ 1e-03 ***
Systolic_min	2.45e-02	2.11e-03	$\ll$ 1e-03 ***	3.76e-02	9.21e-03	$\ll$ 1e-03 ***
Diastolic_min	4.79e-02	3.46e-03	$\ll$ 1e-03 ***	3.05e-02	1.45e-02	3.54e-02 **
Resp rate_min	3.36e-02	2.24e-02	1.35e-01	-3.40e-02	8.42e-02	6.86e-01
Heart rate_min	1.47e-02	2.47e-03	$\ll$ 1e-03 ***	2.65e-02	7.73e-03	$\ll$ 1e-03 ***
Saturation_min	1.03e-01	1.18e-02	$\ll$ 1e-03 ***	2.27e-02	2.90e-02	4.33e-01
Temperature_first	-6.54e-02	2.23e-02	3.38e-03 ***	-9.86e-02	8.73e-02	2.59e-01
Systolic_first	-1.63e-03	7.80e-04	3.70e-02 **	-4.09e-03	3.70e-03	2.69e-01
Diastolic_first	4.83e-03	1.37e-03	$\ll$ 1e-03 ***	-1.32e-02	6.77e-03	5.06e-02 *
Resp rate_first	7.09e-03	9.71e-03	4.65e-01	-7.09e-03	3.54e-02	8.41e-01
Heart rate_first	1.15e-03	9.58e-04	2.29e-01	-8.18e-03	3.68e-03	2.62e-02 **
Saturation_first	-1.86e-02	5.94e-03	1.76e-03 ***	-2.85e-03	1.53e-02	8.52e-01
Temperature_last	-2.74e-01	2.67e-02	$\ll$ 1e-03 ***	3.36e-01	7.92e-02	$\ll$ 1e-03 ***
Systolic_last	2.46e-04	8.37e-04	7.69e-01	-9.15e-03	3.79e-03	1.58e-02 **
Diastolic_last	9.91e-03	1.52e-03	$\ll$ 1e-03 ***	-1.69e-02	6.91e-03	1.43e-02 **
Resp rate_last	-1.46e-02	1.06e-02	1.66e-01	-1.27e-02	3.59e-02	7.23e-01
Heart rate_last	-6.34e-03	1.04e-03	$\ll$ 1e-03 ***	1.25e-02	3.31e-03	$\ll$ 1e-03 ***
Saturation_last	3.77e-02	6.40e-03	$\ll$ 1e-03 ***	-3.15e-02	1.35e-02	1.96e-02 **
Temperature_mean	-1.52e-01	9.58e-02	1.13e-01	7.51e-01	2.95e-01	1.10e-02 **
Systolic_mean	7.56e-03	2.86e-03	8.20e-03 ***	-1.50e-02	1.36e-02	2.71e-01
Diastolic_mean	-3.33e-02	4.89e-03	$\ll$ 1e-03 ***	-1.47e-02	2.29e-02	5.19e-01
Resp rate_mean	-5.11e-02	3.29e-02	1.21e-01	1.51e-01	1.29e-01	2.40e-01
Heart rate_mean	7.86e-03	3.29e-03	1.70e-02 **	7.58e-02	1.07e-02	$\ll$ 1e-03 ***
Saturation_mean	2.75e-02	2.02e-02	1.73e-01	4.51e-02	5.46e-02	4.09e-01
Temperature_SD	9.01e+00	4.44e+00	4.21e-02 **	-4.61e+01	1.09e+01	$\ll$ 1e-03 ***
Systolic_SD	5.00e-02	1.28e-02	$\ll$ 1e-03 ***	1.71e-01	5.81e-02	3.20e-03 ***
Diastolic_SD	2.11e-01	2.27e-02	$\ll$ 1e-03 ***	2.33e-01	1.05e-01	2.73e-02 **
Resp rate_SD	6.86e-02	8.04e-02	3.94e-01	-4.51e-01	2.51e-01	7.29e-02 *
Heart rate_SD	1.35e-02	1.23e-02	2.73e-01	-7.47e-02	4.40e-02	8.94e-02 *
Saturation_SD	8.06e-01	2.21e-01	$\ll$ 1e-03 ***	2.92e-01	6.88e-01	6.72e-01
Temperature_APC	-2.18e+01	4.07e+00	$\ll$ 1e-03 ***	-4.34e+01	1.70e+01	1.06e-02 **
Systolic_APC	-2.68e+00	3.99e-01	$\ll$ 1e-03 ***	-1.02e+00	2.07e+00	6.22e-01
Diastolic_APC	-9.24e-01	3.62e-01	1.07e-02 **	-1.64e-02	1.73e+00	9.92e-01
Resp rate_APC	-1.83e-01	4.92e-01	7.10e-01	1.31e-01	1.58e+00	9.34e-01
Heart rate_APC	-1.79e+00	2.67e-01	$\ll$ 1e-03 ***	-5.56e+00	1.26e+00	$\ll$ 1e-03 ***
Saturation_APC	2.35e+00	2.43e+00	3.34e-01	7.22e+00	7.47e+00	3.34e-01
Temperature_ACPTU	9.91e-01	1.87e-01	$\ll$ 1e-03 ***	1.69e+00	8.98e-01	5.95e-02 *
Systolic_ACPTU	5.99e-02	6.29e-03	$\ll$ 1e-03 ***	9.73e-02	3.66e-02	7.94e-03 ***
Diastolic_ACPTU	1.06e-01	9.37e-03	$\ll$ 1e-03 ***	5.33e-02	5.94e-02	3.69e-01
Resp rate_ACPTU	-3.36e-02	6.48e-02	6.05e-01	-4.59e-01	2.19e-01	3.62e-02 **
Heart rate_ACPTU	9.12e-02	6.93e-03	$\ll$ 1e-03 ***	4.61e-02	3.28e-02	1.59e-01
Saturation_ACPTU	-3.48e-02	4.59e-02	4.48e-01	-2.58e-01	1.37e-01	5.93e-02 *
Temperature_IQR	-1.16e+00	6.48e-02	$\ll$ 1e-03 ***	-8.75e-01	2.26e-01	$\ll$ 1e-03 ***
Systolic_IQR	-7.60e-03	1.91e-03	$\ll$ 1e-03 ***	-1.77e-02	8.69e-03	4.22e-02 **
Diastolic_IQR	-2.19e-02	3.34e-03	$\ll$ 1e-03 ***	-1.64e-02	1.62e-02	3.14e-01
Resp rate_IQR	3.73e-02	2.42e-02	1.23e-01	5.09e-02	8.52e-02	5.50e-01
Heart rate_IQR	-1.13e-02	2.37e-03	$\ll$ 1e-03 ***	-1.61e-02	8.35e-03	5.33e-02 *
Saturation_IQR	-5.01e-02	1.50e-02	$\ll$ 1e-03 ***	3.52e-02	4.57e-02	4.41e-01
Temperature_CV	-1.23e+02	1.63e+02	4.50e-01	1.86e+03	3.99e+02	$\ll$ 1e-03 ***

**Covariate**	**Favourable discharge**			**Deterioration**		
	$\hat{\beta}$	**s.e**	p**-value**	$\hat{\beta}$	**s.e**	p**-value**
Systolic_CV	1.92e+00	1.54e+00	2.14e-01	-8.58e+00	6.87e+00	2.12e-01
Diastolic_CV	-5.56e+00	1.56e+00	$\ll$ 1e-03 ***	-1.17e+01	6.63e+00	7.81e-02 *
Resp rate_CV	-8.47e-01	1.36e+00	5.33e-01	1.21e+01	4.82e+00	1.21e-02 **
Heart rate_CV	2.86e+00	8.92e-01	1.35e-03 ***	2.20e+01	3.45e+00	$\ll$ 1e-03 ***
Saturation_CV	-6.12e+01	2.13e+01	4.10e-03 ***	-4.17e+00	6.23e+01	9.47e-01
Age	-1.79e-03	6.53e-04	6.02e-03 ***	5.01e-02	3.90e-03	$\ll$ 1e-03 ***
Concious_II	-4.16e-01	1.02e-01	$\ll$ 1e-03 ***	1.31e+00	1.91e-01	$\ll$ 1e-03 ***
Concious_III	-4.97e-01	2.91e-01	8.80e-02 *	1.46e+00	2.40e-01	$\ll$ 1e-03 ***
Concious_missing	-2.66e-01	1.95e-02	$\ll$ 1e-03 ***	2.21e-01	1.16e-01	5.61e-02 *
SexFemale	1.42e-01	1.86e-02	$\ll$ 1e-03 ***	-1.80e-01	9.27e-02	5.25e-02 *

Table C: FG-Full estimated coefficients. Significance codes: *** for p-value < 0.01; ** for p-value < 0.05; * for p-value < 0.1.

Covariate	Favourable discharge			Deterioration		
	$\hat{\beta}$	s.e	p-value	$\hat{\beta}$	s.e	p-value
Temperature_max	-9.24e-01	8.10e-02	$\ll$ 1e-03 ***	1.15e-01	1.63e-01	4.80e-01
Systolic_max	-1.24e-02	3.37e-03	$\ll$ 1e-03 ***	-7.85e-03	9.07e-03	3.90e-01
Diastolic_max	-3.34e-02	5.52e-03	$\ll$ 1e-03 ***	1.89e-02	1.39e-02	1.70e-01
Resp rate_max	-4.31e-03	1.96e-02	8.30e-01	2.12e-02 **	6.23e-02	7.30e-01
Heart rate_max	-1.46e-02	3.92e-03	$\ll$ 1e-03 ***	-1.89e-02 **	8.38e-03	2.40e-02 **
Saturation_max	-6.89e-02	2.28e-02	2.50e-03 ***	-7.60e-02	5.07e-02	1.30e-01
Temperature_min	1.37e+00	9.22e-02	$\ll$ 1e-03 ***	-3.03e-01	2.14e-01	1.60e-01
Systolic_min	1.65e-02	3.50e-03	$\ll$ 1e-03 ***	7.45e-03	1.08e-02	4.90e-01
Diastolic_min	3.30e-02	4.94e-03	$\ll$ 1e-03 ***	-7.08e-03	1.63e-02	6.60e-01
Resp rate_min	1.74e-02	2.46e-02	4.80e-01	-1.33e-01	7.65e-02	8.20e-02 *
Heart rate_min	8.42e-03	4.17e-03	4.40e-02 **	-7.51e-04	8.84e-03	9.30e-01
Saturation_min	7.34e-02	1.60e-02	$\ll$ 1e-03 ***	9.91e-02	3.58e-02	5.70e-03 ***
Temperature_first	-1.45e-02	3.01e-02	6.30e-01	-1.47e-01	8.56e-02	8.60e-02 *
Systolic_first	-2.86e-04	9.52e-04	7.60e-01	-1.09e-03	3.69e-03	7.70e-01
Diastolic_first	6.78e-03	1.67e-03	$\ll$ 1e-03 ***	-1.30e-02	6.44e-03	4.30e-02 **
Resp rate_first	1.18e-03	1.13e-02	9.20e-01	-4.37e-02	3.81e-02	2.50e-01
Heart rate_first	3.29e-03	1.18e-03	5.40e-03 ***	-1.14e-02	3.88e-03	3.40e-03 ***
Saturation_first	-1.41e-02	7.84e-03	7.20e-02 *	8.06e-03	1.67e-02	6.30e-01
Temperature_last	-2.45e-01	4.05e-02	$\ll$ 1e-03 ***	3.84e-01	9.04e-02	$\ll$ 1e-03 ***
Systolic_last	1.55e-03	1.20e-03	2.00e-01	-1.20e-02	4.24e-03	4.80e-03 ***
Diastolic_last	1.18e-02	2.11e-03	$\ll$ 1e-03 ***	-1.93e-02	7.36e-03	8.60e-03 ***
Resp rate_last	8.56e-03	1.32e-02	5.20e-01	4.72e-02	3.54e-02	1.80e-01
Heart rate_last	-8.58e-03	1.62e-03	$\ll$ 1e-03 ***	1.67e-02	3.64e-03	$\ll$ 1e-03 ***
Saturation_last	4.10e-02	8.72e-03	$\ll$ 1e-03 ***	-2.13e-02	1.73e-02	2.20e-01
Temperature_mean	-3.35e-01	1.43e-01	1.90e-02 **	8.45e-01	3.43e-01	1.40e-02 **
Systolic_mean	3.01e-03	4.67e-03	5.20e-01	-1.30e-02	1.59e-02	4.10e-01
Diastolic_mean	-2.41e-02	7.94e-03	2.40e-03 ***	1.99e-02	2.66e-02	4.50e-01
Resp rate_mean	-5.10e-02	3.63e-02	1.60e-01	2.18e-01	1.28e-01	8.90e-02 *
Heart rate_mean	4.34e-04	4.82e-03	9.30e-01	6.97e-02	1.19e-02	$\ll$ 1e-03 ***
Saturation_mean	1.64e-02	2.61e-02	5.30e-01	-2.13e-01	6.05e-02	$\ll$ 1e-03 ***
Temperature_SD	5.45e+00	7.48e+00	4.70e-01	-3.91e+01	1.53e+01	1.01e-02 **
Systolic_SD	5.75e-02	2.67e-02	3.10e-02 **	6.41e-02	8.55e-02	4.50e-01
Diastolic_SD	7.70e-02	4.64e-02	9.70e-02 *	-1.65e-02	1.52e-01	9.10e-01
Resp rate_SD	2.07e-01	1.11e-01	6.30e-02 *	-5.62e-01	2.49e-01	2.40e-02 **
Heart rate_SD	-6.45e-03	3.13e-02	8.40e-01	1.48e-01	6.76e-02	2.80e-02 **
Saturation_SD	-3.99e-01	1.29e-01	1.90e-03 ***	1.11e-01	3.25e-01	7.40e-01
Temperature_APC	-1.46e+01	8.03e+00	6.90e-02 *	3.58e+00	1.77e+01	8.40e-01
Systolic_APC	-2.55e+00	6.08e-01	$\ll$ 1e-03 ***	1.84e+00	1.92e+00	3.40e-01
Diastolic_APC	-3.19e-01	5.20e-01	5.40e-01	-8.18e-01	2.68e+00	7.60e-01
Resp rate_APC	-6.19e-01	6.08e-01	3.10e-01	1.80e-01	1.62e+00	9.10e-01
Heart rate_APC	2.64e-01	5.04e-01	6.00e-01	-5.51e+00	1.46e+00	$\ll$ 1e-03 ***
Saturation_APC	-2.76e+00	4.40e+00	5.30e-01	-1.08e+01	8.13e+00	1.80e-01
Temperature_ACPTU	8.96e-01	5.34e-01	9.40e-02 *	-7.06e-01	9.80e-01	4.70e-01
Systolic_ACPTU	4.44e-02	1.19e-02	$\ll$ 1e-03 ***	-2.09e-02	3.55e-02	5.60e-01
Diastolic_ACPTU	1.02e-01	1.48e-02	$\ll$ 1e-03 ***	-2.59e-02	6.84e-02	7.10e-01
Resp rate_ACPTU	1.12e-02	9.95e-02	9.10e-01	-1.23e-02	2.31e-01	9.60e-01
Heart rate_ACPTU	5.66e-02	1.49e-02	$\ll$ 1e-03 ***	9.52e-03	3.67e-02	8.00e-01
Saturation_ACPTU	2.61e-01	8.08e-02	1.30e-03 ***	2.26e-01	1.59e-01	1.60e-01
Temperature_IQR	-7.98e-01	1.05e-01	$\ll$ 1e-03 ***	4.05e-02	2.44e-01	8.70e-01
Systolic_IQR	-6.05e-03	2.86e-03	3.40e-02 **	-6.07e-03	1.00e-02	5.40e-01
Diastolic_IQR	-1.34e-02	4.92e-03	6.30e-03 ***	-1.90e-02	1.65e-02	2.50e-01
Resp rate_IQR	2.65e-02	2.54e-02	3.00e-01	-8.84e-02	8.50e-02	3.00e-01
Heart rate_IQR	-6.83e-03	3.64e-03	6.00e-02 *	-1.11e-02	8.96e-03	2.10e-01
Saturation_IQR	-4.18e-02	2.03e-02	4.00e-02 **	-3.52e-02	5.05e-02	4.90e-01
Temperature_CV	-6.91e+01	2.75e+02	8.00e-01	1.47e+03	5.58e+02	8.60e-03 ***

Covariate	Favourable discharge			Deterioration		
	$\hat{\beta}$	**s.e**	***p*-value**	$\hat{\beta}$	**s.e**	***p*-value**
Systolic_CV	-1.70e+00	3.04e+00	5.80e-01	-5.46e+00	9.50e+00	5.70e-01
Diastolic_CV	-1.30e-01	3.12e+00	9.70e-01	2.70e+00	1.04e+01	7.90e-01
Resp rate_CV	-2.55e+00	1.68e+00	1.30e-01	1.14e+01	4.78e+00	1.70e-02 **
Heart rate_CV	-5.43e-01	1.58e+00	7.30e-01	1.64e+01	4.02e+00	$\ll$ 1e-03 ***
Saturation_CV	-1.42e+02	3.62e+01	$\ll$ 1e-03 ***	-1.62e+02	7.51e+01	3.10e-02 **
Age	-8.07e-03	1.04e-03	$\ll$ 1e-03 ***	3.45e-02	3.46e-03	$\ll$ 1e-03 ***
Concious_II	-9.06e-01	1.92e-01	$\ll$ 1e-03 ***	1.24e+00	2.27e-01	$\ll$ 1e-03 ***
Concious_III	-2.79e+00	4.41e-01	$\ll$ 1e-03 ***	1.42e+00	3.05e-01	$\ll$ 1e-03 ***
Concious_missing	-2.36e-01	2.99e-02	$\ll$ 1e-03 ***	2.98e-01	1.10e-01	6.90e-03 ***
SexFemale	1.63e-01	2.68e-02	$\ll$ 1e-03 ***	-3.32e-01	8.94e-02	$\ll$ 1e-03 ***

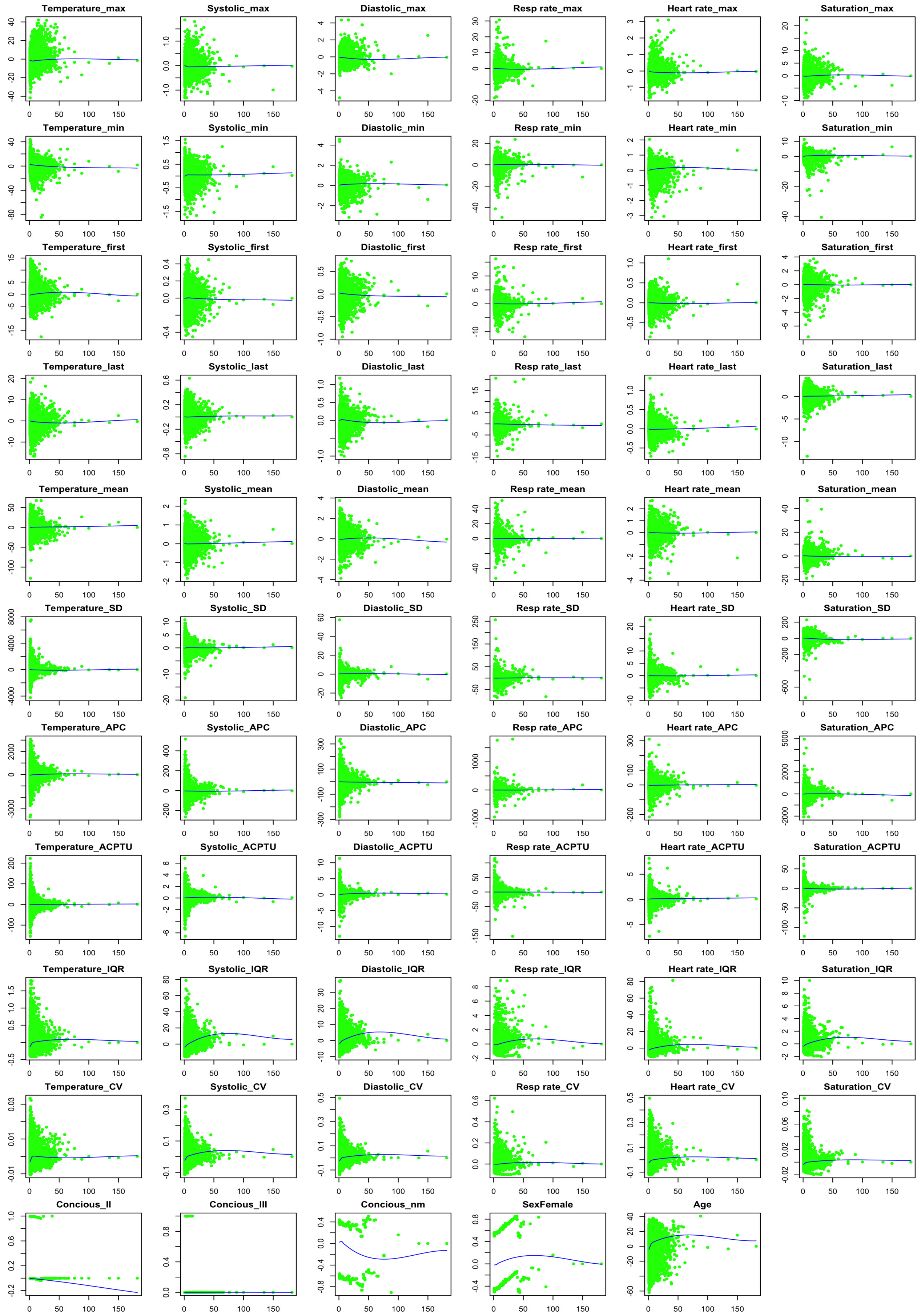

Fig S2. Scaled Schoenfeld residuals from the CSC-Full model for favorable discharge, for all transversal statistics across all vital signs. Y-axis: residual values; X-axis: Length of Stay (in days). Residuals are displayed as filled green points, and the adjusted spline estimated using a loess smoother is represented by the blue line.

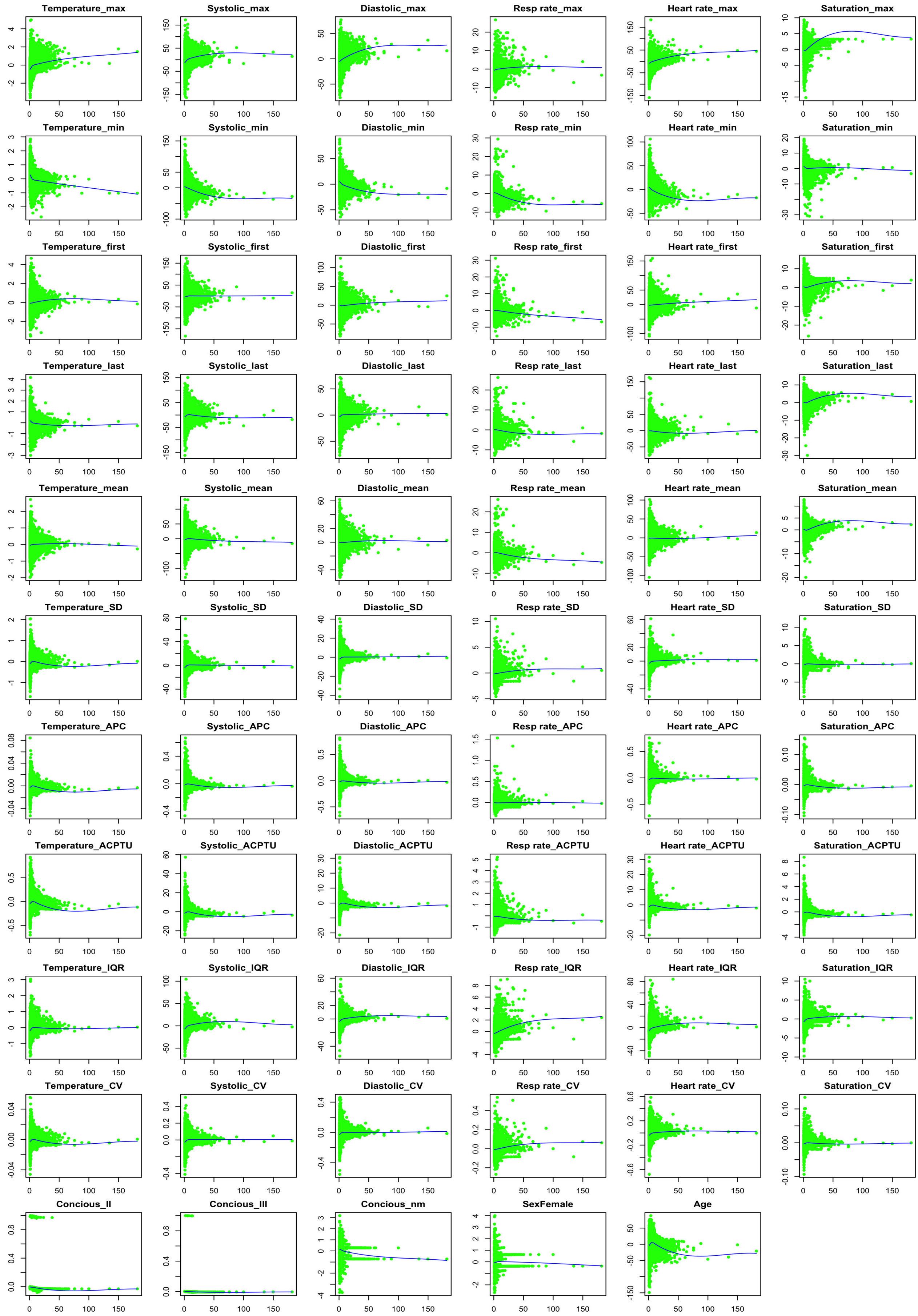

Fig S3. Scaled Schoenfeld residuals from the FG-Full model for favorable discharge, for all transversal statistics across all vital signs. Y-axis: residual values; X-axis: Length of Stay (in days). Residuals are displayed as filled green points, and the adjusted spline estimated using a loess smoother is represented by the blue line.

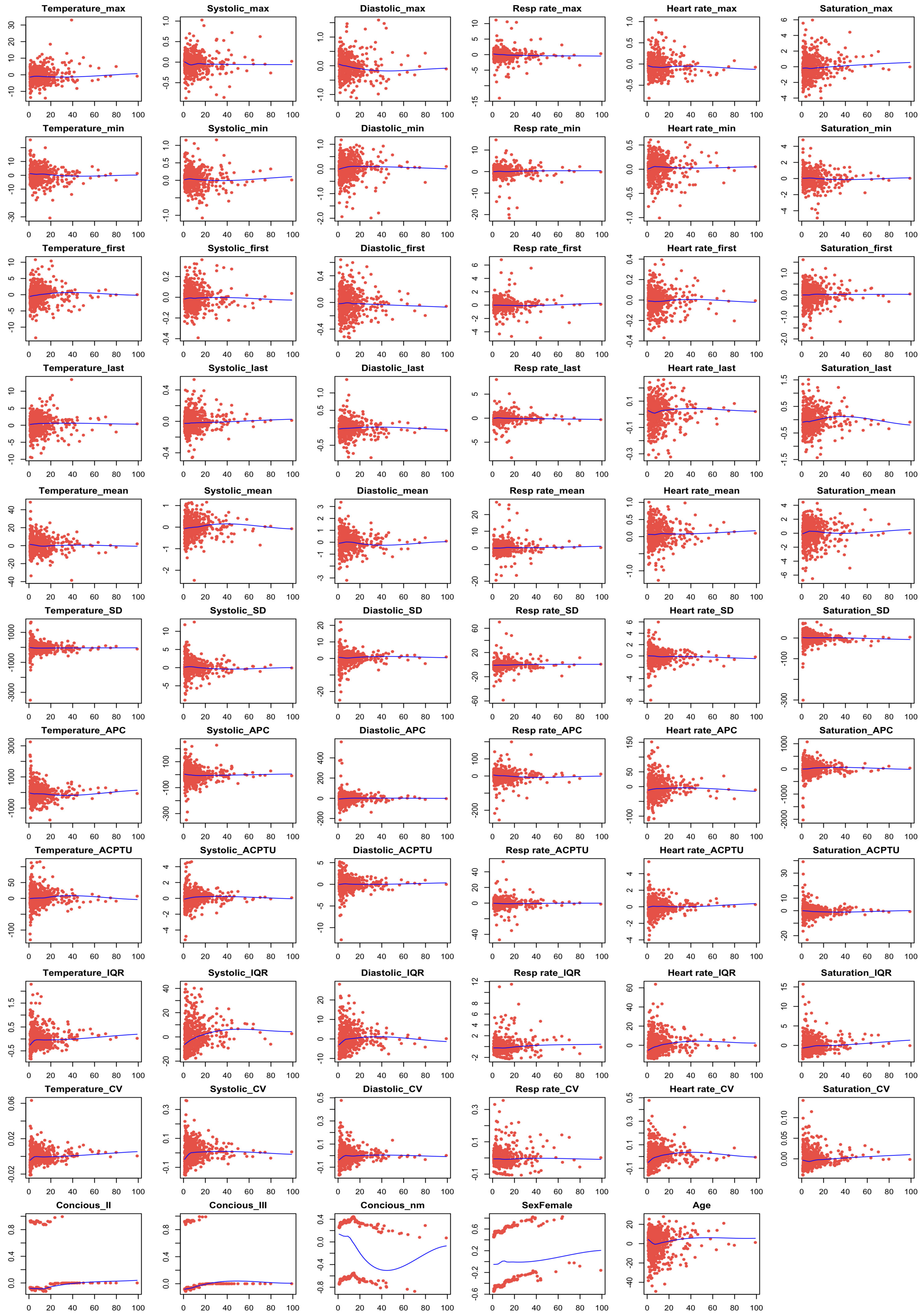

Fig S4. Scaled Schoenfeld residuals from the CSC-Full model for favorable discharge, for all transversal statistics across all vital signs. Y-axis: residual values; X-axis: Length of Stay (in days). Residuals are displayed as filled green points, and the adjusted spline estimated using a loess smoother is represented by the blue line.

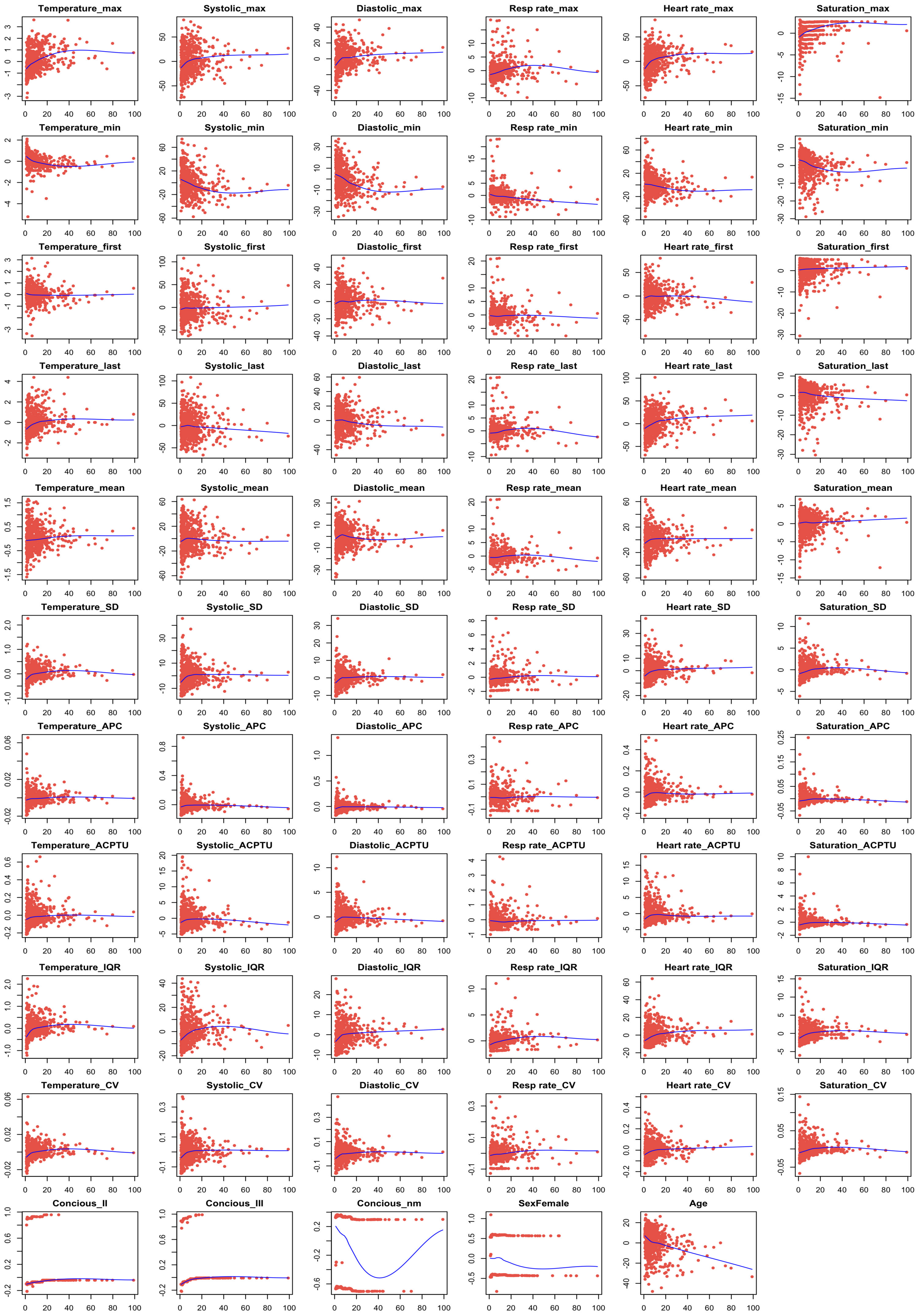

Fig S5. Scaled Schoenfeld residuals from the FG-Full model for favorable discharge, for all transversal statistics across all vital signs. Y-axis: residual values; X-axis: Length of Stay (in days). Residuals are displayed as filled green points, and the adjusted spline estimated using a loess smoother is represented by the blue line.

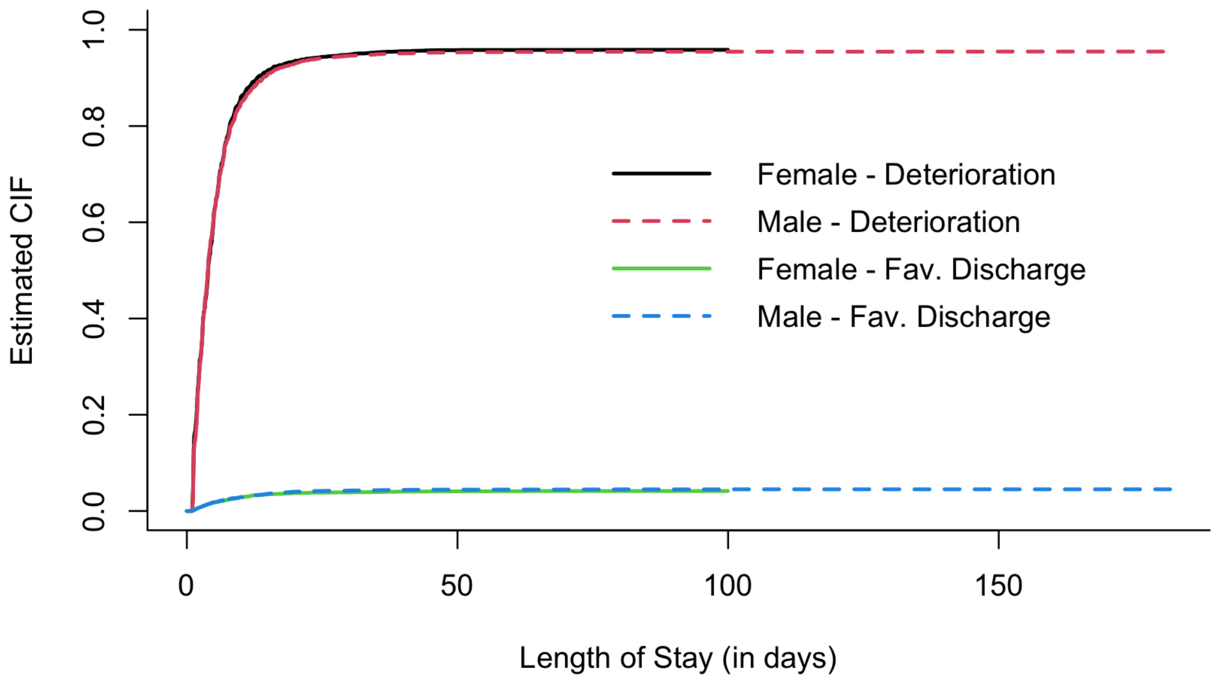

Fig S6. Non-parametric estimates of cumulative incidence functions using the Aalen-Johansen estimator, stratified by sex.

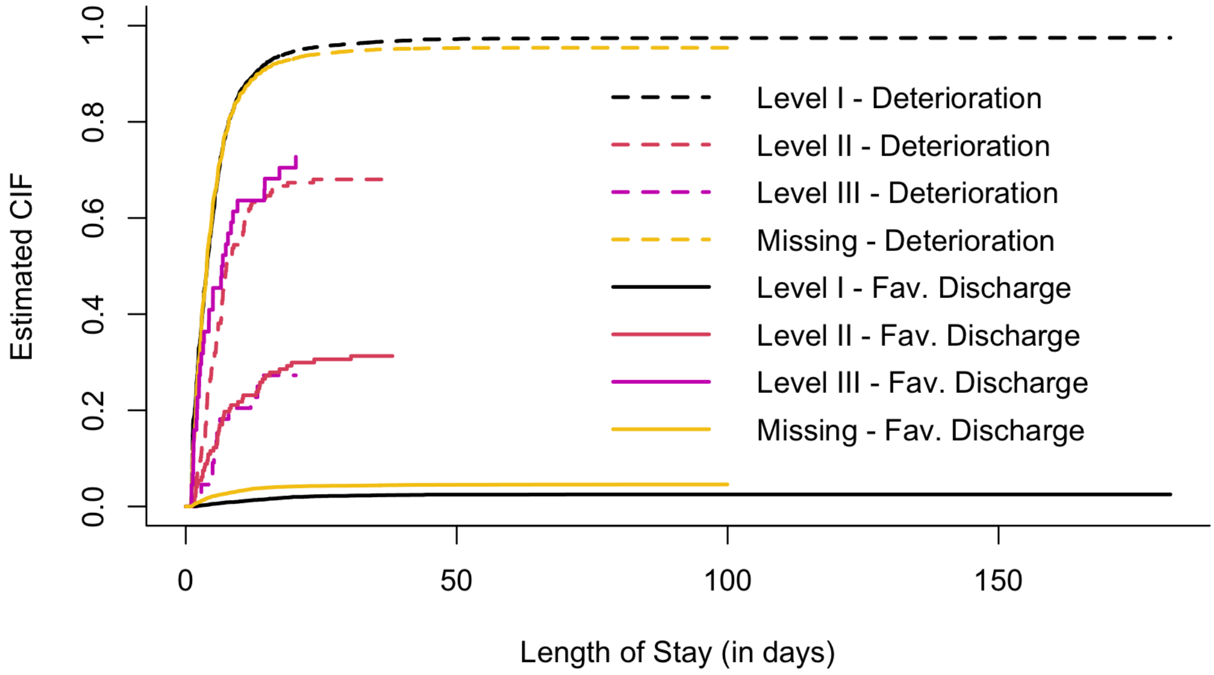

Fig S7. Non-parametric estimates of cumulative incidence functions using the Aalen-Johansen estimator, stratified by consciousness levels.

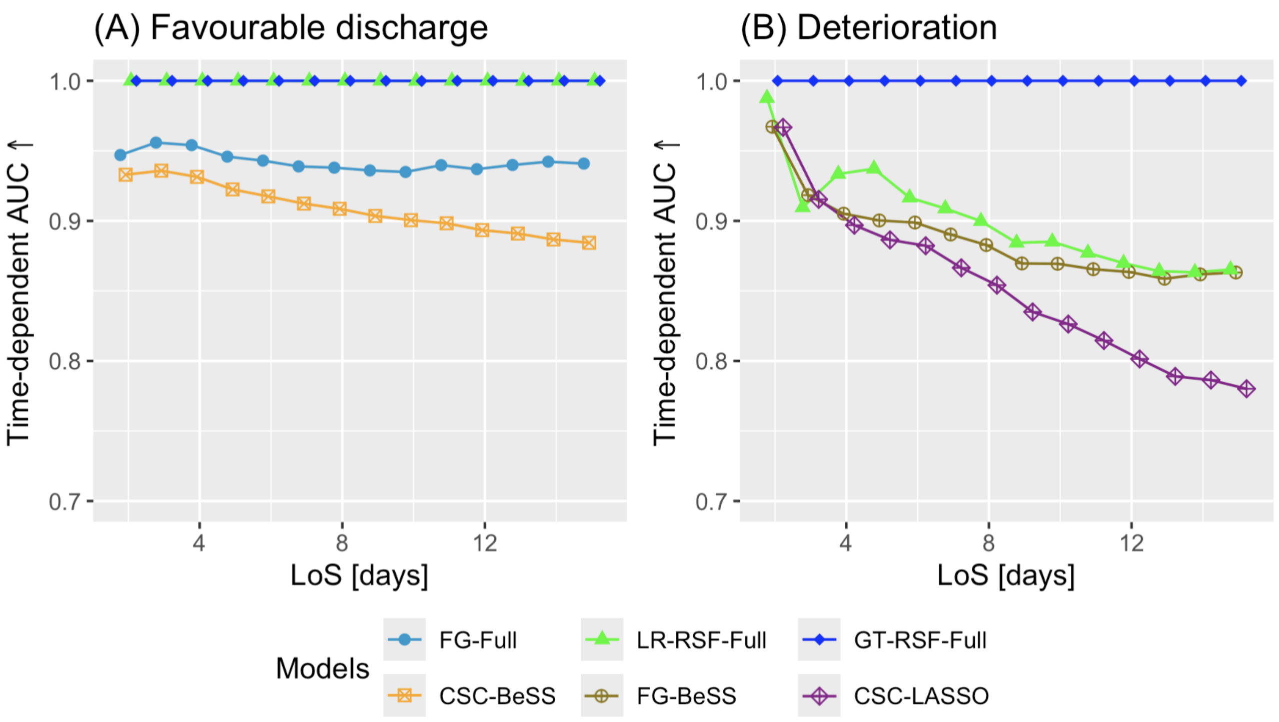

Fig S8. Time-dep AUC↑ performance for the best case for each type of CR model.

S.C. Hyperparameters

Table D. Parameters and hyperparameters used for imputation, variable selection, and CR modelling steps. The specific R functions and the values used are detailed.

Step	Method/Model	Parameter/Hyperparameter	Value	R function
**Imputation**	MICE	Number of imputed datasets	5	mice::futuremice
		Number of iterations	10	
		Method	pmm	
	BPCA	Principal components	20	pcaMethods::pca
		Method	bpca	
		Scaling	uv	
	NIPALS	Principal components	20	pcaMethods::pca
		Method	nipals	
		Scaling	uv	
**Variable selection**	LASSO	Family	cox	glmnet::cv.glmnet
		Loss function	C	
	BeSS	Family	cox	BeSS::bess
		Method	sequential	
		Model size	40	BeSS::bess.one
**Modelling**	Tune RSF	Hyperparameter tuning	–	randomForestSRC::tune
	Trees RSF	Number of trees	100	randomForestSRC::rfsrc
	GT-RSF-Full	Nodesize	1	
		Variables to split	21	
	GT-RSF-Null	Nodesize	4	
		Variables to split	1	
	LR-RSF-Full Fav. Discharge	Nodesize	1	
		Variables to split	17	
	LR-RSF-Null Fav. Discharge	Nodesize	4	
		Variables to split	1	
	LR-RSF-Full Deterioration	Nodesize	3	
		Variables to split	50	
	LR-RSF-Null Deterioration	Nodesize	3	
		Variables to split	1	
	GT-RSF-LASSO	Nodesize	6	
		Variables to split	12	
	LR-RSF-LASSO Fav. Discharge	Nodesize	10	
		Variables to split	12	
	LR-RSF-LASSO Deterioration	Nodesize	1	
		Variables to split	32	
	GT-RSF-BeSS	Nodesize	3	
		Variables to split	14	
	LR-RSF-BeSS Fav. Discharge	Nodesize	1	
		Variables to split	21	
	LR-RSF-BeSS Deterioration	Nodesize	1	
		Variables to split	7	
